# Developing a dual VEGF/PDL1 inhibitor based on high-affinity scFv heterodimers as an anti-cancer therapeutic strategy

**DOI:** 10.1038/s41598-023-39076-8

**Published:** 2023-07-24

**Authors:** Noam Tzuri, Ksenia M. Yegodayev, Ofra Novoplansky, Moshe Elkabets, Amir Aharoni, Niv Papo

**Affiliations:** 1 Avram and Stella Goldstein-Goren Department of Biotechnology Engineering and The National Institute of Biotechnology in the Negev, Ben-Gurion University of the Negev, 8410501 Beer-Sheva, Israel; 2 The Shraga Segal Department of Microbiology, Immunology, and Genetics, Ben-Gurion University of the Negev, 8410501 Beer-Sheva, Israel; 3 Department of Life Sciences and The National Institute of Biotechnology in the Negev, Ben-Gurion University of the Negev, 8410501 Beer-Sheva, Israel

**Keywords:** Oncogene proteins, Protein design, Drug development

## Abstract

Cancer progression is enhanced by the interaction of programmed death-ligand 1 (PDL1), which is associated with inhibition of the immune response against tumors, and vascular endothelial growth factor (VEGF), which inhibits immune cell activity while inducing angiogenesis and proliferation of cancer cells. Dual inhibition of PDL1 and VEGF may therefore confer a synergistic anti-cancer therapeutic effect. We present a novel strategy for developing a therapeutic that simultaneously binds and inhibits both PDL1 and VEGF. We generated a bi-specific protein, designated DuRan-Bis, comprising a single chain variable fragment (scFv)-based inhibitor of PDL1 fused to an scFv-based inhibitor of VEGF, with the latter being attached to an Fc fragment. We found that DuRan-Bis binds to both PDL1 and VEGF with high affinity. Compared to treatments with mono-specific proteins, alone or in combination, the DuRan-Bis chimera showed superior inhibition of the proliferation of glioblastoma cells. In comparison to treatment with immune cells alone, a combination of immune cells with DuRan-Bis decreased the viability of head and neck cancer cells. To the best of our knowledge, this study is the first to use a single polypeptide chain scFv-scFv-Fc scaffold for engineering a high-affinity bi-specific inhibitor of PDL1 and VEGF.

## Introduction

Bi-specific antibodies are a class of engineered immunoglobulin (IgG)-like or antibody fragment (Fab)-like proteins that combine different antigen-binding elements in a single construct. The superiority of bi-specific antibodies over mono-specific antibodies, which target only a single molecule, lies in: (1) their ability to simultaneously block two different pathways with unrelated or overlapping pathogenic functions, (2) their enhanced binding specificity and binding efficacy (avidity) that derives from engaging with multiple cell-surface antigens or a combination of cell-surface and soluble antigens, and (3) their lower development and production costs vs. two different mono-specific antibodies used in combination therapy^1,2^. However, the advantages of IgG-like bi-specific antibodies, comprising heterodimers in which each component has a different antigen specificity are offset by several well-known limitations, particularly the random pairing of the variable domains of heavy (V_H_) and light (V_L_) chains, which leads to the production of various mispaired IgG species in addition to the single functional bi-specific antibody (thus giving a low yield)^3,4^.

The above 'mispairing' issues can be prevented by preparing bi-specific antibodies from single chain variable fragment (scFv)-based scafolds, in which the V_L_ of the variable domain and the corresponding V_H_ are fused in tandem, thus preventing any V_L_–V_H_ mismatch. For example, a bi-specific antibody can be prepared by fusing an scFv to the Fc fragment at the C-terminus of IgG (i.e., IgG-scFv) or by fusing two scFvs via a peptide linker (i.e., a tandem scFv) to generate a molecule that binds one antigen at the N-terminus of the tandem molecule and a similar (or different) antigen at the C-terminus^5–7^. Indeed, a bi-specific tandem scFv comprising two different scFvs with different antigen specificities has been developed to target both the CD3 receptor on T cells and tumor-associated antigens on cancer cells. This type of antibody construct, which constitutes the basis of the novel BiTE® (bi-specific T-cell engager) molecules, has been applied in cancer immunotherapy to re-target T cells to tumor cells in the tumor microenvironment (TME)^8^. Nevertheless, despite their promise, a critical limitation of BiTEs is their small size and consequent rapid clearance from the blood circulation^5^.

A slightly different type of antibody construct, a bi-specific scFv-scFv-Fc fusion molecule, has been prepared with an Fc domain at the C-terminus^5^. The Fc fragment increases the solubility and stability of the protein, improves its pharmacokinetic profile, and enhances avidity via Fc dimerization, leading to enhanced local (near target) antibody concentrations^9,10^. It has been shown, for example, the bi-specific inhibitor to a disintegrin and metalloproteinase 17 (ADAM17) and TNF-like ligand 1A (TL1A) can bind to ADAM17 located on the cell surface and exhibit up to 100-fold higher inhibition of TL1A-induced cytokine secretion and apoptosis of TF-1 cells, relative to the mono-TL1A inhibitor^11^. An additive or synergistic pharmacological advantage may thus be derived by strongly binding a cell-anchored/soluble antigen pair that simultaneously agonizes and/or antagonizes different biochemical pathways.

An important candidate target pair, comprising soluble and membrane-bound targets, for bi-specific antibody therapy would be vascular endothelial growth factor (VEGF) and programmed death-ligand 1 (PDL1): VEGF, when overexpressed, inhibits immune cell activity and promotes the proliferation of cancer cells, angiogenesis and the formation of abnormal vasculature, whereas PDL1, which is expressed on the surface of cancer cells, is an immune checkpoint molecule that inhibits the immune response against tumors cells by interacting with the PD1 receptor that is expressed on immune cell membranes. PDL1 and VEGF, each alone but especially in combination, have recently been identified as critical modules for cancer progression. For example, VEGF, which binds to its receptor, VEGFR, that is expressed on T cells, leads to upregulation of PD1 on the T cells and to a reduction in T-cell-mediated cancer cell cytotoxicity^12–14^. Moreover, a positive correlation between VEGF and PDL1 expression in cancer cells (glioma, renal cell carcinoma, or head and neck squamous cell carcinoma) has also been observed^15,16^.

Different immune checkpoint inhibitors targeting PDL1, e.g., atezolizumab or durvalumab, or anti-angiogenesis agents targeting VEGF, e.g., bevacizumab, have therefore been developed to target this axis. Bevacizumab is a recombinant humanized monoclonal antibody that blocks all isoforms of human VEGF with high efficacy. The approval of bevacizumab by the FDA, in 2004, for the treatment of metastatic colorectal cancer^17^, was followed in 2006 by the approval of the VEGF inhibitor, ranibizumab, a monoclonal Fab generated from the same parent mouse antibody as bevacizumab and used for the treatment of neovascular (wet) age-related macular degeneration^18^. However, monotherapy blockage of VEGF signaling has been associated with cancer disease relapse, increased invasion, and the formation distant metastases due to the upregulation of compensation and adaptation mechanisms. It thus became apparent that a hyperinvasive and metastatic phenotype was accompanied by increased hypoxia in the TME^19,20^, which is known to induce up-regulation of PDL1 as a compensation mechanism^21^. The failure of single-agent anti-VEGF therapy—due to compensation by other pathways (e.g., PDL1)—indicates that combination with a different anti-tumor therapy could be an effective direction for the development of therapeutics. Suitable candidates for combination with anti-VEGF therapy are immune checkpoint inhibitors, such as anti-PDL1 monoclonal antibodies. These antibodies have shown strong antitumor activity against a variety of solid tumors, e.g., atezolizumab for renal cell carcinoma, avelumab for urothelial carcinoma, and durvalumab for urothelial carcinoma or non-small cell lung cancer^22–27^. Nevertheless, the activity of PDL1-inhibitors is limited, being dependent on the recruitment of T cells to the TME, which is impaired in different types of cancer and in different disease stages due to tumor vasculature abnormalities^21,28–31^. In addition, some cancers may acquire resistance to anti-PD1/PDL1 therapies over time, resulting in T-cell exhaustion by virtue of different mechanisms, including enhancement of PD1 or VEGF expression, with the latter manifesting as suppression of the cytotoxicity of immune cells. These limitations strongly suggest that efficacious treatment will require an additional strategy in combination with PD1/PDL1 inhibition^32,33^.

Indeed, in a variety of models, a combination of therapeutic antibodies against PDL1 and VEGF has shown synergistic clinical benefit in tumor control vs. anti-VEGF or anti-PDL1 treatment alone or vs. anti-VEGF treatment combined with cytotoxic chemotherapeutic agents^13,14,34,35^. Taken together, the above-described studies reinforce the understanding that combined therapy targeting PDL1 and VEGF has significant potential in cancer treatment. Nevertheless, combined therapy also has limitations, including interactions between two different drugs and high development and production costs; these limitations could, however, be overcome by generating a single bi-specific agent. There have indeed been several attempts to create bi-specific therapies against PDL1 and VEGF, such as the production of bi-specific antibodies targeting both PDL1 and VEGF by fusing together anti-PDL1 antibody with the soluble extracellular domain of vascular endothelial growth factor receptor 1 (VEGFR1), but the process suffered from byproduct generation due to V_L_ shuffling^36^.

To address the above-described issues, we set out to generate and test a high-affinity bi-specific inhibitor of VEGF and PDL1 that was constructed on an scFv-scFv-Fc scaffold. To this end, we first tested the apparent affinity of scFvs that were derived from different antibodies targeting PDL1 and VEGF, and from those we chose the scFvs with highest affinity to the targets. We then designed and constructed a bi-specific inhibitor from a durvalumab-derived scFv (targeting PDL1) and a ranibizumab-derived Fab fragment (targeting VEGF) fused to an Fc fragment. Thereafter, we tested the bi-specific protein binding to VEGF and PDL1 and showed that it did indeed simultaneously target human cell-expressed PDL1 and purified soluble human VEGF with high affinity. Finally, we tested the inhibitory activity of the bi-specific protein and showed that it displayed superior activity in inhibiting glioblastoma proliferation vs. the inhibitory activity of the mono-specific treatments, each alone, or of two mono-specific inhibitors combined. In addition, the bi-specific inhibitor combined with immune cells impaired the viability of head and neck cancer cells, probably due to inhibitor-enhanced increased cytotoxicity of the immune cells.

To the best of our knowledge, our study is the first to use a single polypeptide chain scFv-scFv-Fc scaffold for engineering a high-affinity bi-specific inhibitor of VEGF and PDL1. The scFv-scFv-Fc scaffold of the bi-specific inhibitor obviates the problem of the formation of undesired by-products associated with bi-specific IgG-like antibodies due to mismatch pairing of their four polypeptide chains, while maintaining all the advantages of an Fc fragment (improved solubility, pharmacokinetic profile, and binding efficacy).

## Materials and methods

### Binding of yeast surface displayed (YSD) scFv clones to soluble PDL1 and VEGF_121_

The DNA sequences of three PDL1-targeting scFvs derived from the three different full-length antibodies, atezolizumab, avelumab and durvalumab, and hence designated At, Av, and Du, respectively, were synthesized by Integrated DNA Technologies (Coralville, IA, USA). The DNA sequence of the VEGF-targeting scFv that was derived from the Fab antibody fragment, ranibizumab, and hence designated Ran, was also synthesized by Integrated DNA Technologies. The pCTCON plasmid^37^ was linearized with Xho1-HF and Nhe1-HF restriction enzymes (New England Biolabs, Ipswich, MA, USA), and the plasmid together with one of the four scFv inserts were transformed by homologous recombination into competent *Saccharomyces cerevisiae* EBY100 yeast cells (Thermo Fisher Scientific, Waltham, MA, USA). The correct sequence was confirmed by DNA sequencing in the Genetics Unit of the National Institute for Biotechnology in the Negev (NIBN), Ben-Gurion University of the Negev (BGU), Israel.

A single yeast colony for each of the four scFvs was used for each yeast display binding experiment, as previously described^37^. Briefly, the yeast cells were grown in an incubator at 30 °C for one day in SC-U-T (for Ura and Trp selection) liquid medium, containing 2% w/v glucose, 0.67% w/v yeast nitrogen base, 0.1% v/v histidine, 2% v/v leucine, and 10% v/v dropout solution of all the necessary amino acids (except histidine and leucine). On the following day, the cells were diluted into an SGCAA induction medium, containing 2% w/v galactose, 0.67% w/v yeast nitrogen base, 0.5% w/v Bacto™ Casamino acids, 0.856% w/v sodium phosphate monobasic monohydrate, and 0.54% w/v disodium phosphate, and incubated overnight at 30 °C. On the third day, the cells were collected and washed with PBSA buffer [phosphate buffered saline (PBS) + 0.1% bovine serum albumin (BSA)]. Thereafter, to determine the apparent affinity of the scFv to PDL1 or VEGF, the yeast-displayed cells were incubated for 30 min at room temperature with solutions of biotinylated ligands, i.e., either human PDL1 or human VEGF_121_ (a VEGF isoform lacking heparin-binding ability; ACRObiosystems, Newark, DE, USA) at different concentrations, and then washed with PBSA. An allophycocyanin (APC)-streptavidin conjugate (Jackson ImmunoResearch, West Grove, PA, USA) diluted 1:50 in PBSA was then used to determine the binding of the biotinylated ligands to the scFvs upon 30 min of incubation. The expression of each YSD scFv (having a c-myc tag fused to the C-terminus scFv) was also determined by incubating the yeast samples with an FITC-labeled anti-c-myc antibody (Miltenyi Biotec, Bergisch Gladbach, Germany) at a ratio of 1:50 c-myc to PBSA for 30 min at room temperature. The controls were untreated cells and APC- or FITC labeled cells without ligand. The samples were analyzed using an Accuri C6 flow cytometry analyzer (BD Biosciences, San Diego, CA, USA). Since preliminary experiments showed ligand depletion at low ligand concentrations, we used reaction volumes of 500 µL and concentrations of 0.005, 0.01, 0.02, 0.05, 0.1, 0.2, 0.5, 1, 2, 10, and 20 nM of PDL1. For VEGF_121_, the concentrations were 1, 2, 5, 7.5, 10, 15, 20, 25, 30, 40, 50, 75 and 100 nM in reaction volumes of 50 µL. For generating binding titration curves, each measurement was normalized first to the expression level of each clone and then to the highest binding level of the same clone, which was set as 1. For generating a plot of normalized binding vs. ligand concentration, the control sample containing fluorescent dyes but no ligand was set as zero. To obtain the apparent K_D_ value for each scFv-target protein, the data was fitted to a 1:1 Langmuir kinetic binding model by using GraphPad Prism software (GraphPad software, San Diego, CA, USA).

### Construction of mono- and bi-specific genes in pFUSE

The pFUSE-hIgG1e3-Fc2 plasmid was designed to include the mono-specific proteins (Du, Av or Ran) or the bi-specific protein. Note that the Du- and Ran-containing scFvs are designated here as DuRan-Bis, and the Av- and Ran-containing scFvs are designated as AvRan-Bis. Both the mono- and bi-specific proteins were constructed to include the interleukin-2 (IL-2) signal peptide, an Fc fragment, and a His tag, as shown in Supplementary Fig. S1.

For generating the mono-specific genes (Du, Av or Ran), the plasmid containing the Fc fragment was amplified to insert the scFv (Du, Av or Ran) at the 5' end of the Fc fragment. The amplification was performed with KAPA HiFi DNA polymerase (Merck KGaA, Darmstadt, Germany) using primers with homology to the plasmid (primers A in Supplementary Table S1). Thereafter, the scFv DNA inserts were amplified twice—the first amplification for adding a linker in the 3' end of the scFv, and the second amplification for creating homology to the Fc fragment on the plasmid. The first PCR amplification was performed using KOD Hot Start DNA polymerase (Merck KgaA) and suitable primers (primers B1 for Av, B2 for Du, and B3 for Ran; Supplementary Table S1). The second amplification was performed on the scFvs gene from the previous step using KOD DNA polymerase with suitable primers (primers C1, C2, and C3 for Av, Du, and Ran, respectively; Supplementary Table S1). Each Av, Du, or Ran scFv PCR product was cloned into the open pFUSE vector containing the Fc fragment and a His-tag according to the protocol of the Gibson assembly method (New England Biolabs, Ipswich, MA, USA).

For generating the bi-specific gene, DuRan-Bis, the plasmid that contained scFv Ran with the Fc domain and His-tag from the previous step was amplified and linearized by PCR to enable insertion of anti-PDL1 scFv Av or Du at the 5' end of Ran scFv. The amplification was performed using PCR with a KAPA HiFi DNA polymerase using primers that were homologous to the plasmid (primers E in Supplementary Table S1). PCR amplification of anti-PDL1 scFvs Av and Du was performed using KOD DNA polymerase with forward primers with homology to pFUSE and reverse primers with homology to the Ran scFv gene (primers F1 for Av and F2 for Du in Supplementary Table S1). Thereafter, the Du or Av scFv PCR product was cloned into the linearized pFUSE vector containing the Ran scFv, the Fc fragment, and a His-tag, according to the protocol of the Gibson assembly method. The plasmids containing the mono-specific proteins (Av, Du or Ran) or the bi-specific proteins (DuRan-Bis or AvRan-Bis) were then transformed into electro-competent *Escherichia coli* cells, which were grown overnight on LB low-salt agar plates containing 25 µg/ml Zeocin. The plasmids were then extracted from the bacteria using NucleoSpin Plasmid EasyPure Mini kit (Macherey–Nagel) and sequenced in the Genetics Unit, NIBN, BGU, Israel.

### Cell culture

Suspension-adapted FreeStyle™ human embryonic kidney HEK293-F cells (American Type Culture Collection Manassas, VA, USA) were routinely cultured in GIBCO FreeStyle™ 293 expression medium (Thermo Fisher Scientific) at densities of 0.1 × 10^6^ to 3.0 × 10^6^ cells/ml )more than 90% of the cells were viable) in disposable Erlenmeyer tissue culture flasks with vented caps (TriForest, Irvine, CA, USA) at 125–135 rpm on an orbital shaker incubator (37 °C, 8% CO_2_). Cell density and viability were determined using a Countess II FL Cell Viability Analyzer (Thermo Fisher Scientific) according to the manufacturer's protocols.

HEK 293-T cells [American Type Culture Collection (ATCC), Manassas, VA, USA] and head and neck SCC47^38^ cells (kindly supplied by Reidar Grénman, Department of Otorhinolaryngology—Head & Neck Surgery, Turku University, Finland) were grown in Dulbecco’s modified Eagle’s medium (Biological Industries, Beit Haemek, Israel). Glioblastoma U87MG cells (ATCC) were grown in MEM medium (Biological Industries). These two media were supplemented with 10% FBS, 1% l-glutamine, and 1% penicillin/streptomycin (Biological Industries), referred to below as "plus supplements". The cells were placed in an incubator at 37 °C and 5% CO_2_.

### Construction of the bi-specific heterodimer in pHAGE containing IRES and BFP

The lentivector pHAGE vector was designed to include the bi-specific AvRan-Bis or DuRan-Bis gene. An IL-2 signal peptide and the bi-specific sequence were fused to the 5' end of the internal ribosome entry site (IRES), followed by the blue fluorescent protein (BFP) sequence, with the IRES being used to separate the bi-specific AvRan-Bis or DuRan-Bis from BFP, which was used to quantify the amount of bi-specific protein expressed. The pHAGE lentivector plasmid that contained the IRES and BFP sequences was digested with Not1 and BamH1 (Thermo Fisher Scientific) restriction enzymes, followed by purification with a PCR purification kit. Thereafter, the IL-2 signal peptide and the bi-specific AvRan-Bis or DuRan-Bis protein constructs were amplified from a pre-formed lentiviral F9 plasmid containing IL-2 and the bi-specific gene. The amplification was performed using a PCR reaction with KOD DNA polymerase with primers that were homologous to the pHAGE plasmid (primers G in Supplementary Table S1). Thereafter, the PCR product was cloned into the open pHAGE plasmid using the protocol of the Gibson assembly method, followed by transformation into electro-competent *E. coli*. Colony PCR for Gibson assembly and plasmid insertion validation was performed using ALLin™ Red Taq Mastermix, and colonies positive for the above sequences were transferred to a culture medium containing ampicillin-LB (25 µg/mL) and grown overnight at 37 °C. The pHAGE lentiviral plasmid bearing the bispecific gene was extracted from the bacteria using NucleoSpin Plasmid EasyPure Mini kit and sequenced (Genetics Unit, NIBN, BGU, Israel).

### Generation of HEK293T cells overexpressing AvRan-Bis or DuRan-Bis

The pHAGE lentiviral vector used for transfection of the HEK293T cells was prepared from the packaging HIV expression plasmids, pGag-Pol (5 μg), pTAT (3 μg), and pREV (5 μg), the envelope protein pVSV-G (6 μg), and the pHAGE lentiviral plasmid containing AvRan-Bis or DuRan-Bis (20 μg) in a total volume of 500 µL of medium without supplements. This DNA mixture was incubated for 15 min at room temperature in 500 µL of a solution comprising 120 µL of polyethyleneimine (PEI) (Polysciences, Warrington, PA, USA) transfection reagent plus 380 µL of medium without supplements, which gave a concentration of 1 μg DNA mixture in 3 μL of PEI. One day before transfection, HEK293T cells were seeded in a 10-cm plate in DMEM plus supplements and grown to 60% confluency before transfection. The above-described DNA-PEI mixture was added dropwise to these HEK293T cells after their resuspension in 4 mL of DMEM without supplements.

Five hours post transfection, the transfection medium was replaced with fresh medium plus supplements. The packaged recombinant lentivirus containing pHAGE with AvRan-Bis or DuRan-Bis was harvested from the supernatant of the cell culture at 48 h post transfection, centrifuged at 1200 rpm for 5 min, and filtered through a 0.45-μm filter. The filtrate containing the recombinant lentivirus was stored at -80 °C until used for subsequent experiments. To prepare the frozen recombinant lentivirus for use in experiments, the frozen samples were concentrated at 21,000 rpm for 2 h at 4 °C.

To infect HEK293T cells with the pHAGE lentiviral plasmid containing AvRan-Bis or DuRan-Bis that was mentioned above, HEK293T were seeded at a concentration of 2 × 10^5^ in a 6-well plate. On the following day, the cells were then infected as follows. Most of culture medium of the cells was removed, leaving only 1 mL of medium in each well. Then, to each well was added 250 μL of the concentrated AvRan-Bis or DuRan-Bis-containing lentivirus described above. On the following day, the transduction medium was replaced with DMEM plus supplements. After several days of growth, cells were harvested by centrifugation and then resuspended in PBS buffer (containing 10% FBS) for FACS sorting at the Ilse Katz Institute for Nanoscale Science and Technology, BGU, Israel. Transduced cells (1.0 × 10^6^) expressing the 20% highest level of BFP were sorted, centrifuged, and seeded into fresh DMEM plus supplements in a 10-cm plate.

### Production and purification of recombinant proteins

Du and Ran were produced in mammalian HEK293F cells maintained in FreeStyle 293 expression medium. The HEK293F cells were transiently transformed with 1 µg/mL of each of the pFUSE DNA plasmids containing Du or Ran scFvs (generated as described above in the section "Construction of mono- and bi-specific genes in pFUSE") according to the FreeStyle 293 manual, using the GeneTranIII transfection reagent (Biomiga, San Diego, CA, USA).

A stable HEK293T cell culture containing DuRan-Bis, which was generated by infection (as described above in the section "Construction of the bi-specific heterodimer in pHAGE containing IRES and BFP"), was seeded at 20% confluency into DMEM plus supplements. On the next day, the medium was discarded, the cells were washed twice with DMEM without supplements, and fresh FreeStyle medium was added to each flask, which was then incubated for 72 h at 37 °C and 5% CO_2_. The supernatant was harvested after 3 days, centrifuged for 5 min at 1000 rpm, and filtered.

The Du, Ran, and DuRan-Bis proteins that were secreted to the culture medium were concentrated, and the medium was replaced with binding (and wash) buffer (50 mM Tris pH 8.0, 200 mM NaCl, and 50 mM imidazole) using the ÄKTA flux system with a 5-kDa cutoff (GE Healthcare Systems, Boston, MA, USA). The protein solution was rotated for 1 h at 4 °C with Protino® Ni–NTA Agarose nickel beads (Macherey–Nagel) for purification. The beads were loaded into the column, washed with wash buffer, and then eluted with elution buffer (50 mM Tris pH 8.0, 200 mM NaCl, and 500 mM imidazole). To determine protein purity, SDS-PAGE analysis was performed using a 10% polyacrylamide gel under reducing conditions, and the bands were visualized by staining with Instant Blue (Abcam, Cambridge, UK). The eluted protein was dialyzed overnight at 4 °C, and the buffer was exchanged with PBS using GeBaFlex-tube dialysis kit (GeBa, Yavne, Israel). Concentration of the purified protein was determined by UV–visible absorbance at 280 nm, using a NanoDrop spectrophotometer (DeNovix Inc, Wilmington, DE, USA), with an extinction coefficient (ϵ_280_) of 15,9475 M^−1^·cm^−1^ for DuRan-Bis, 93,905 M^−1^**·**cm^−1^ for Du, and 101,355 M^−1^**·**cm^−1^ for Ran. The purified proteins were aliquoted and stored at -80 °C until further use.

### Cell binding assay

Affinity titration analysis and apparent K_D_ measurements were performed as previously described^39^. In brief, U87MG cells (5 × 10^4^) endogenously expressing PDL1 were incubated for 1 h on ice with 2 mL of purified Du protein at concentrations of 0.001, 0.02, 0.05, 0.1, 0.2, 0.5, 1, 2 or 5 nM or with 2 mL of purified DuRan-Bis protein at concentrations of 0.001, 0.02, 0.05, 0.1, 0.2, 0.5, 1, 2, 5, 10, 20 or 50 nM diluted in FACS buffer containing PBSA. Cells were then washed with FACS buffer and incubated for 40 min in 40 μL of 1:50 dilution of APC-conjugated anti-His tag antibody (Abcam, Cambridge, UK) on ice in the dark. Cells were washed, resuspended in FACS buffer, and analyzed in a Diva flow cytometer (BD Biosciences, San Diego, CA, USA). The experiment was performed in triplicate, and error bars in the figures represent the standard error of the mean (SEM).

To generate the concentration-dependent affinity titration curves from the geometric mean measurement of each sample, we subtracted the negative control (cells incubated without DuRan-Bis or Du proteins but with anti-His antibody) and then normalized the signal to the highest binding signal of the same individual sample, which was set as 1 and to the negative control, set as 0. A 1:1 Langmuir kinetic binding model was implemented for all the binding plots using GraphPad Prism software (GraphPad software), and apparent K_D_ values for Du and DuRan-Bis proteins were calculated.

To confirm dual binding of DuRan-Bis to PDL1 and VEGF_121_, U87MG cells (5 × 10^5^) endogenously expressing PDL1 were incubated for 1 h on ice with 50 μL of either purified Du or DuRan-Bis protein, diluted to 100 nM in FACS PBSA buffer. The cells were then washed in FACS buffer and incubated with 50 μL of biotinylated VEGF_121_, 100 nM, for 1 h on ice. Thereafter, the cells were washed in FACS buffer and incubated in the dark for 40 min in 50 μL of 1:50 dilution of APC-streptavidin targeting the biotinylated VEGF_121_. Cells were washed and resuspended in FACS buffer, and the samples were then analyzed using a Diva flow cytometer.

### Surface plasmon resonance analysis of Du, Ran and DuRan-Bis binding to purified PDL1 and VEGF_121_

The equilibrium binding affinities of Du, Ran, or DuRan-Bis inhibitors for VEGF_121_ and PDL1 targets were determined by surface plasmon resonance (SPR) on a Biacore S2000 instrument (Biacore, Uppsala, Sweden). In these experiments, purified rhVEGF_121_ (R&D Systems, Minneapolis, MN, USA), and purified human PDL1 (ACRObiosystems) were used. After standard amine-coupling activation of the CM5 sensor chip using a solution of N-hydroxysuccinimide/1-ethyl-3-(3-dimethylaminopropyl)carbodiimide (NHS/EDC), 10 nM PDL1 or VEGF_121_ (in sodium acetate at pH 4.6) was immobilized in the appropriate channel to reach 812 RU for VEGF121 or 760 RU for PDL1. Thereafter, to generate the binding sensorgrams, various concentrations of Du or DuRan-Bis proteins (analytes) in PBST were injected into the channels at a flow rate of 30 µL/min for 2 min to minimize mass transport effects. The sample compartment temperature was 12 °C, and sample injection temperature was 25 °C. Kinetic parameters were derived from data sets acquired according to single-cycle kinetics. Each run consisted of 9 consecutive analyte injections at 2, 4, 8, 16, 32, 64, 128, 256 and 512 nM Ran (as the analyte in a VEGF_121_-immobilized channel); 0.2 nM, 0.33, 0.55, 0.926, 1.543, 2.572, 4.286, 7.144, and 11.9 nM Du (as the analyte in a PDL1-immobilized channel); 2, 4, 8, 16, 32, 64, 128, 256, and 512 nM DuRan-Bis (as the analyte in a VEGF_121_-immobilized channel); or 50, 62, 78, 98, 122, 153, 192, 240, and 300 nM DuRan-Bis (as the analyte in a PDL1-immobilized channel). Dissociation of the Du, Ran, or DuRan-Bis from the VEGF_121_ or PDL1 immobilized on the CM5 chip was measured for 900 s. The dissociation of DuRan-Bis from PDL1 was followed by a regeneration step with 4 mM NaOH at 10 µL/min for 30 s. The dissociation of Ran from VEGF_121_ was followed by a regeneration step with 20 mM NaOH at 10 µL/min for 30 s. The dissociation rate constant (kd), the association rate constant (ka), and the dissociation equilibrium constant (K_D_) were calculated with Biacore BIAevaluation S200 Software 1.1 (Biacore, Inc., Piscataway, NJ, USA). To achieve statistical significance, we confirmed that the χ2 values were at least 10% or lower than the Rmax values in each experiment. To evaluate the simultaneous binding of DuRan-Bis to VEGF_121_ and PDL1, we injected 254 nM DuRan-Bis as the first analyte (where VEGF_121_ was already immobilized on the sensor chip) followed by an injection of 500 nM PDL1 as the second analyte.

### XTT proliferation assay

An XTT assay was performed to evaluate the effect of DuRan-Bis, Du, and Ran on the proliferation of U87MG glioblastoma cells. First, cells were seeded into a 96-well plate at a density of 5 × 10^3^ per well in MEM medium containing 10% FBS and incubated overnight at 37 °C. Then, the medium was replaced with starvation medium containing 0.5% FBS in the absence or presence of 1 nM human VEGF_121_ and in the absence or presence of DuRan-Bis at 1 nM, 5 nM, 20 nM and 40 nM. A separate experiment, with a similar protocol, was also conducted in the absence or presence of 1 nM human VEGF_121_, but with 100 nM of Du, Ran, a mixture of Du and Ran, or DuRan-Bis. The plates were incubated for 48 h at 37 °C, 5% CO_2_. After 48 h, cell proliferation was quantified by using an XTT kit (Biological Industries), according to the manufacturer's instructions. Absorbance for each sample was measured at 475 nm, with an additional measurement at 660 nm for background subtraction for each well. Wells containing medium alone were considered as the negative control, and absorbance was measured at 475 nm using an ELISA reader infinite M200 (Tecan Trading AG, Switzerland). The experiment was performed in triplicate; error bars in the figures represent the SEM; and statistical significance was determined by column statistics and Student’s t-test, with *P* < 0.05 being considered significant. The results were normalized to the samples containing no VEGF_121_ and no Du, Ran or DuRan-Bis inhibitors.

### Cytotoxicity assays

To evaluate the effect of DuRan-Bis, Du, and Ran on the viability of SCC47 head and neck cancer cells in the presence of immune cells, a cytotoxicity assay was performed as previously described ^7^, with the following modifications. On day one of the experiment, effector cells, namely, peripheral blood mononuclear cells (PBMCs), from a healthy donor were cultured for 48 h at 37 °C with 5% CO_2_ in RPMI 1640 medium supplemented with 10% human serum, 1% sodium pyruvate, 1% l-glutamine, 1% MEM non-essential amino acids, 1% penicillin/streptomycin, 1% HEPES, 200 IU/mL recombinant human IL-2, and 50 ng/mL anti-human CD3 (OKT3). On the second day, target cells, namely, SCC47 cells, infected with green fluorescent protein (GFP), as previously described^40^, were seeded into a 96-well plate at a density of 1 × 10^4^ per each well in DMEM medium containing 10% FBS and incubated overnight at 37 °C. On the third day (after 48 h of effector cell culture), the target cells in the 96-well plate were subjected to the following 20 different treatments: the first five treatments comprised 50 μL of the culture medium of the effector cells (without the effector cells) plus 50 μL of DMEM medium alone or in presence of 12.5 nM Du, Ran, or a mixture of Du and Ran or DuRan-Bis. The remining 15 treatments comprised 50 μL of culture medium with effector cells (3 × 10^4^, 5 × 10^4^ or 1 × 10^5^ cells representing 1:3, 1:5 or 1:10 target to effector cell ratio, respectively) plus 50 μL of DMEM medium alone or in the presence of 2.5, 6.25 or 12.5 nM DuRan-Bis. In addition, 5 × 10^4^ effector cells were also exposed to treatments with 12.5 nM Du, Ran, or a mixture of Du and Ran. The plate was incubated for 48 h at 37 °C with 5% CO_2_. The plate was photographed using a live cell imaging JuLi™ stage (NanoEnTek Inc, Seoul, Korea) immediately after adding the treatments and again after 48 h to monitor the amount of GFP in SCC47 cells as an indicator of cell viability. The experiment was performed in triplicate and the images were analyzed using Qupath. Statistical significance was determined by column statistics and Student’s t-test, with *P* < 0.05 considered as significant. Error bars in the figures represent SEM.

### In-vivo toxicity assay

A toxicity study was performed using 8-week-old NOD scid gamma (NSG™) female mice. DuRan-Bis (1.25 mg/kg) was dissolved in PBS and administered by intraperitoneal (i.p.) injection (n = 6). Control mice were injected with vehicle (n = 5). All mice were weighed before treatment (day 1) and 7 days post treatment, i.e., at the end of the experiment. At that time, the animals were sacrificed, blood was collected from each mouse by cardiac puncture, and serum was isolated by centrifugation (15 min at 2000 g). For the treated group, every two serum samples were pooled, giving a total of three samples for biochemistry analysis (Biochemical Laboratory, Soroka Medical Center, Beer-Sheva, Israel). Hepatic damage was assessed in terms of serum levels of alanine aminotransferase and alkaline phosphatase, and renal damage was determined according to levels of serum urea and creatinine. Livers, lungs, kidneys, intestines and spleens of all the mice were weighed at the end of the experiment.

## Results and discussion

### Binding of scFvs to PDL1 and VEGF_121_

**Figure 1:**
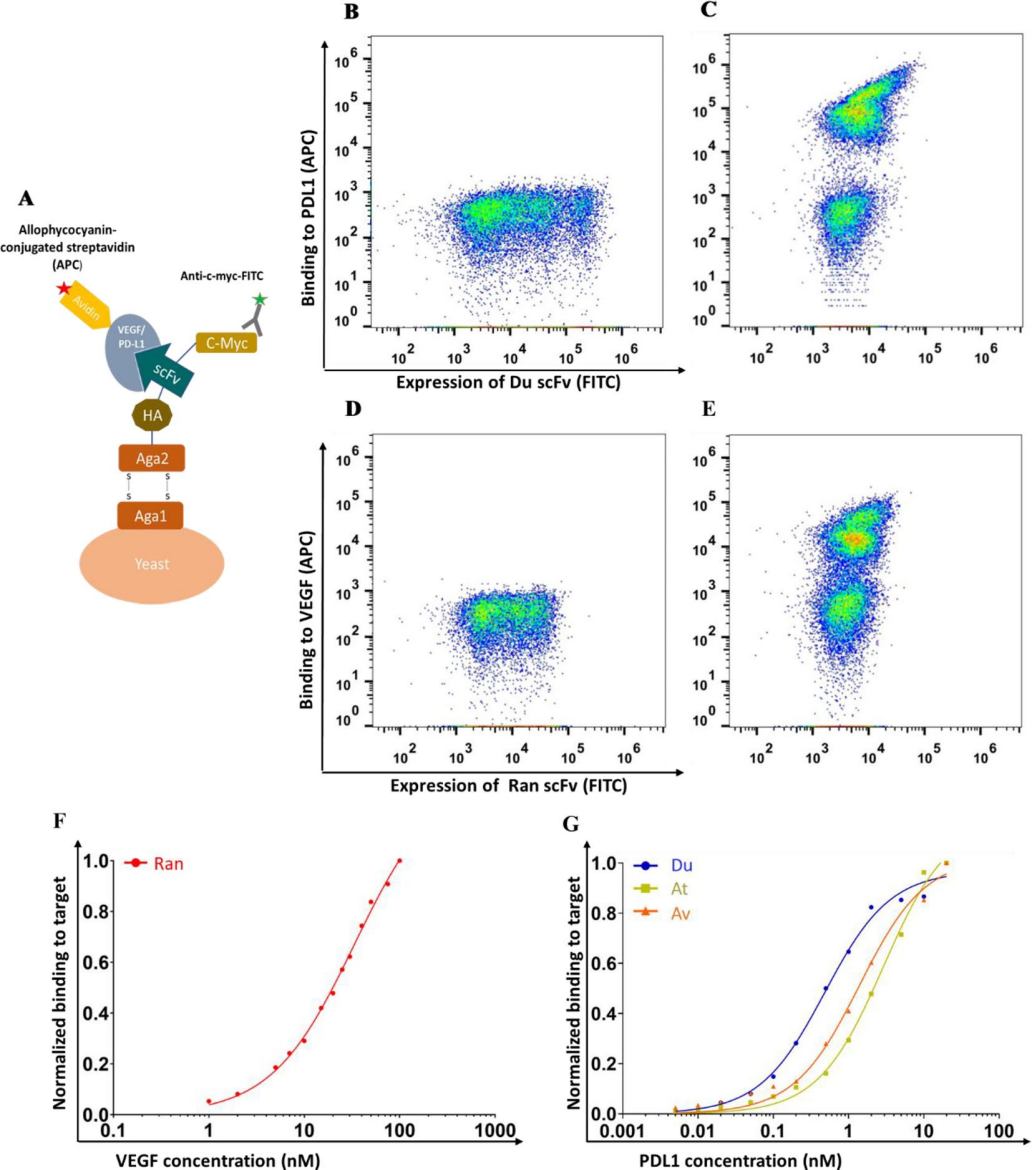
Single chain variable fragment (scFv) binding to programmed death-ligand 1 (PDL1) or vascular endothelial growth factor (VEGF). (**A**) Schematic representation of the yeast surface display (YSD) system. The scFv fused to Aga2p expressed in yeast is disulfide-bonded to Aga1p, which is covalently bound to the yeast cell wall. Expression of the scFv variant was detected by an anti-c-myc-FITC antibody, and binding was determined using either biotinylated-PDL1 or biotinylated-VEGF_121_, followed by allophycocyanin (APC)-conjugated streptavidin. (**B**–**E**) Flow cytometry measurement of the expression of YSD Du scFv (B) or its binding to 25 nM soluble PDL1 (C), and expression of YSD Ran scFv (D) or its binding to 25 nM soluble VEGF_121_ (**E**). (**F**, **G**) YSD affinity titration curves for the anti-VEGF Ran scFv (F; red dots), anti-PDL1 Du scFv (G; blue dots), At scFv (G; dark yellow squares) and Av scFv (G; orange triangles). Binding was determined by using flow cytometry analysis and different concentrations of VEGF (1 to 100 nM) or PDL1 (0.005 to 20 nM). The binding of each sample was normalized to its own expression level and then to the highest binding signal exhibited by the same scFv clone. The data was fitted to a 1:1 Langmuir kinetic binding model to yield the apparent K_D_ value for each scFv–target protein pair.

In this study, we aimed to generate bi-specific single polypeptide chain agents by fusing together two different scFvs (derived from two different monoclonal antibodies, one targeting PDL1, and the other, VEGF) along with an Fc fragment. First, we sought to test whether each scFv, derived from a larger antibody or an antibody fragment, would indeed bind to its target molecule. To do so, we used yeast surface display (YSD) binding titrations, followed by flow cytometry analysis. The scFvs used here were derived from durvalumab, avelumab, and atezolizumab (designated Du, Av, and At, respectively), which are antibodies targeting PDL1^22–24,41^, and ranibizumab (designated Ran), which is a Fab antibody fragment that targets VEGF^42^. All four scFvs, which were expressed in EBY100 yeast cells as part of the YSD system (Fig. 1A), showed strong binding to the relevant target—either PDL1 or VEGF—as demonstrated by affinity titrations (Fig. 1B–E). As expected, our results showed that the Ran scFv binds strongly to VEGF_121_ in the nanomolar range (apparent K_D_ = 33.430 ± 1.693 nM, Fig. 1F) and Du, Av, and At bind to PDL1 in the low nanomolar range (apparent K_D_ values of 0.483 ± 0.043 nM, 1.392 ± 0.145 nM, and 2.905 ± 0.256 nM, respectively, Fig. 1G). Since the Du and Av scFvs showed lower apparent K_D_ values than the At scFv, we continued with the former two scFvs—along with the Ran scFv—in the subsequent experiments. As an aside, we note, for purposes of comparison, that Cembrola et al. found an apparent K_D_ of 4.55 nM when testing the affinity of an anti-PDL1 scFv by using a binding method similar to ours^43^.

### Design and purification of mono- and bi-specific binders

Mono-specific binders targeting PDL1 were generated by fusing Av or Du scFvs to an Fc fragment, and a mono-specific binder targeting VEGF was generated by fusing Ran scFv to an Fc fragment. Bi-specific binders simultaneously targeting both PDL1 and VEGF, designated DuRan-Bis and AvRan-Bis, were generated by fusing Du or Av scFvs, respectively, with Ran scFv and an Fc fragment. The IgG1 Fc fragment was included because, when fused to proteins, it enhances their stability and in vivo half-life by increasing their size and hence retarding their renal clearance and because it facilitates the recycling of the protein fused to the Fc via the neonatal Fc receptor^10^. In addition, the Fc fragment is known to mediate effector functions, such as antibody-dependent cell-mediated cytotoxicity (ADCC) ^10^, by binding to several types of immune cells, such as NK cells, thereby bringing them into the proximity of the antibody's target cells. Since PDL1 can also be expressed on activated T cells, in which case ADCC can promote an attack on activated immune cells, a durvalumab Fc-domain previously been engineered to eliminate ADCC^44^. Thus, in our study we also used an engineered Fc fragment (that is included in the pFUSE vector) to prevent ADCC. The mono- and bi-specific proteins were sub-cloned into a pFUSE mammalian expression vector, which contains an IL-2 signal peptide responsible for protein secretion outside the cell (at the N-terminus), the engineered FC fragment, and a 6 × His tag that is used for protein purification and detection (at the C-terminus) (Supplementary Fig. S1).

Since transiently transfected HEK293T cells gave low yields of the bi-specific DuRan-Bis and AvRan-Bis proteins, it was first necessary to generate stable HEK293T cells expressing high levels of the proteins. To this end, the genes of the bi-specific proteins were transformed into a mammalian vector, namely, a pHAGE vector containing an IRES sequence and a BFP marker gene that facilitates the detection and quantification of the bi-specific protein expression in cells^45^. (We note that the BFP marker is not part of the final protein and has no effect on the binding and on the biological activity.) For this purpose, a lentivirus containing the pHAGE plasmid was generated and used to infect HEK293T cells. The cells were then sorted using FACS to isolate the cells that showed the 20% highest level of protein expression by using BFP as a marker (Supplementary Fig. S2), such that stable cell cultures expressing the AvRan-Bis or DuRan-Bis bi-specific proteins were generated. The mono-specific proteins, Av, Du, and Ran, were produced in transiently transfected HEK293F cells. The mono-specific protein Av was not stable and yields were very low in large-scale production experiments, and thus we decided to continue working with the DuRan-Bis bi-specific protein and its mono-specific controls, Du and Ran. Of note, the overall yields of DuRan-Bis, Ran, and Du were 1.57, 0.66, and 8.16 mg/L culture medium, respectively. As expected, when the three proteins were run on SDS-PAGE gel under reducing conditions, the size of the bi-specific protein DuRan-Bis was approximately 90 kDa and the size of the two mono-specific proteins Du and Ran was approximately 60 kDa (Supplementary Fig. S3).

### DuRan-Bis binds with high affinity and specificity to both recombinant PDL1 and recombinant VEGF_121_

**Figure 2:**
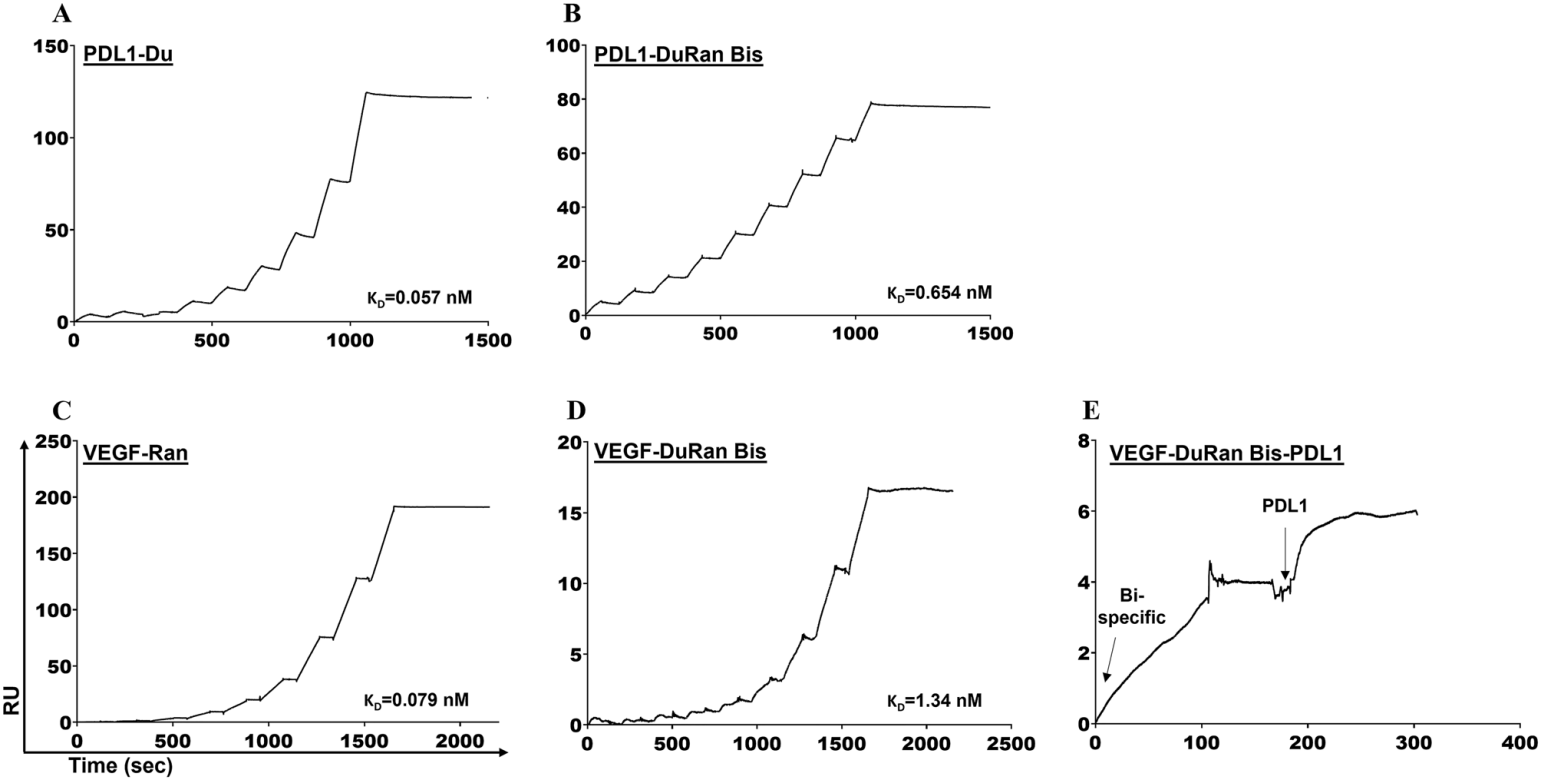
Single-cycle kinetics analysis of surface plasmon resonance (SPR) sensorgrams for binding analysis of the different purified protein complexes. (**A**, **B**) Binding to PDL1 of the mono-specific protein Du at concentrations of 0.2, 0.33, 0.55, 0.93, 1.54, 2.57, 4.29, 7.14, and 11.9 nM (A) and the bi-specific protein DuRan-Bis at concentrations of 50, 62, 78, 98, 122, 153, 192, 240, and 300 nM (B). (**C**,** D**) Binding to VEGF_121_ of the mono-specific protein Ran at concentrations of 2, 4, 8, 16, 32, 64, 128, 256 and 512 nM (**C**) and the bi-specific protein DuRan-Bis at concentrations of 2, 4, 8, 16, 32, 64, 128, 256 and 512 nM (**D**). (**E**) Binding of the bi-specific protein DuRan-Bis (254 nM) to immobilized VEGF, followed by its binding to PDL1 (500 nM). The data was fitted to a 1:1 Langmuir kinetic binding model to yield the K_D_ value for each protein pair. RU—response unit.

The ability of the mono- and bi-specific proteins to bind PDL1 and VEGF_121_ was determined by using SPR. In that experiment, PDL1 or VEGF_121_ was immobilized on a CM5 sensor chip as the ligand, and the proteins Du, Ran, or DuRan-Bis were allowed to flow over the ligand. Kinetic parameters were derived according to single-cycle kinetics. The binding sensorgrams were fitted to a 1:1 Langmuir model, from which the affinity constant (K_D_) was determined (Fig. 2A–D). This analysis revealed that DuRan-Bis bound strongly to PDL1 (K_D_ = 0.654 nM, Fig. 2B) and VEGF_121_ (K_D_ = 1.340 nM, Fig. 2D), but the binding was nonetheless weaker than that of the mono-specific proteins, Du and Ran (Du bound to PDL1 with K_D_ 0.057 nM, and Ran bound to VEGF_121_ with K_D_ 0.079 nM, Fig. 2A and C, respectively). This difference in the affinity between the bi- and mono-specific proteins could be attributed to the reduced accessibility of each individual scFv in the bi-specific protein to its target ligand. The affinity of Ran to recombinant VEGF_121_ and Du to recombinant PDL1 was approximately 10 times higher than that of their parental full-length antibodies ranibizumab (K_D_ ~ 0.99 nM to VEGF_121_)^46^ and durvalumab (K_D_ of 0.831 nM to PDL1)^47^. This difference in affinity could be due to the smaller size of the mono-specific Du and Ran proteins, which resulted in less steric hindrance. We nonetheless stress that even though the affinity of DuRan-Bis appeared to be lower than that of the mono-specific Du and Ran proteins, its affinity still remained very high in the low nano-molar range. Furthermore, it was previously shown in cell-based assays that binding of bi-specific proteins to cell membrane receptors increases the local concentration of the bi-specific protein and thus enhances the binding to the other (soluble) protein that is also targeted^11^. We therefore believe that DuRan-Bis would still have an advantage over the mono-specific proteins in the cellular environment, even though its affinity for its targets was lower than that of the mono-specific proteins (as measured in an in-vitro binding experiment).

Another perspective may be obtained by examining the affinity between PDL1 and its receptor, PD1. According to the literature^48^, the kinetic constants for the binding of PDL1 to PD1 are K_on_ (the association rate constant) = 1.84 × 10^5^ M^−1^ × s^−1^, K_off_ (the dissociation rate constant) = 1.44 s^−1^, and K_D_ (the affinity constant) = 8.2 μM, where K_D_ = K_off_/K_on_. These values indicate that the affinity of PDL1 for its receptor (K_D_ of 8.2 μM) is significantly lower than that for DuRan-Bis (K_D_ of 0.654 nM) or Du (K_D_ of 0.057 nM). Thus, the differences in K_D_ between PD1, Du, and DuRan-Bis may be attributed to much lower K_off_ values for Du and DuRan-Bis (K_off_ 1.69 × 10^−5^ s^−1^ and 3.84 × 10^−5^ s^−1^, respectively) than for PD1 (K_off_ 1.44 s^−1^), which means that our proteins Du and DuRan-Bis dissociate from PDL1 far more slowly than PD1.

The binding between VEGF_121_ and its cognate receptor, VEGFR2, has affinity values of K_on_ = 5.47 × 10^6^ M^−1^ × s^−1^, K_off_ = 3.0 × 10^−3^ s^−1^, and K_D_ = 5.5 nM, as previously determined^48^, which means that that the affinity of VEGF_121_ to VEGFR2 (K_D_ 5.5 nM) is approximately 60 times lower than the affinity of Ran to VEGF_121_ (K_D_ 0.079 nM) and 4 times lower than the affinity of DuRan-Bis to VEGF_121_ (K_D_ 1.340 nM). These differences in K_D_ values are due to the smaller K_off_ for Ran and DuRan-Bis (K_off_ 1.06 × 10^−6^ s^−1^ and 1.39 × 10^–5^ s^−1^, respectively) than for VEGFR2 (K_off_ 3.0 × 10^−3^ s^−1^) from complexes with VEGF_121_, which means that the dissociation of VEGF_121_ from Ran and DuRan-Bis is slower than its dissociation from VEGFR2. These lower dissociation rates for DuRan-Bis, Du, and Ran from both PDL1 and VEGF_121_, in comparison to the dissociation rates (K_off_) of PDL1 and VEGF from their native cognate receptors, could reduce availability of the ligands for binding to their receptors. The potential of DuRan-Bis to compete with and inhibit the functions of VEGF and PDL1 finds support in studies showing that Ran and Du proteins (derived from ranibizumab and durvalumab, respectively) bind to the receptor binding site of the natural ligands (agonists) PDL1 and VEGF, respectively^42,49^.

In addition, we performed an experiment in which the VEGF_121_ ligand, immobilized on the chip, was exposed sequentially to 254 nM DuRan-Bis as the first analyte flowing over the chip and then to 500 nM PDL1 as a second analyte (Fig. 2E). The bi-specific DuRan-Bis protein engaged successfully with PDL1, VEGF_121_, or both of them together, which confirms its ability to efficiently bind PDL1 and VEGF_121_ simultaneously. As a control, we immobilized VEGF_121_ and examined its interaction with PDL1 or with Du, the mono-specific PDL1 binder, but, as expected, no binding was observed between PDL1 and VEGF_121_ or between Du and VEGF_121_. Taken together, these findings show that DuRan-Bis binds strongly to its targets and can engage both of them simultaneously.

### DuRan-Bis binds with high affinity to endogenous PDL1 expressed on U87MG cells and simultaneously engages with recombinant VEGF

**Figure 3:**
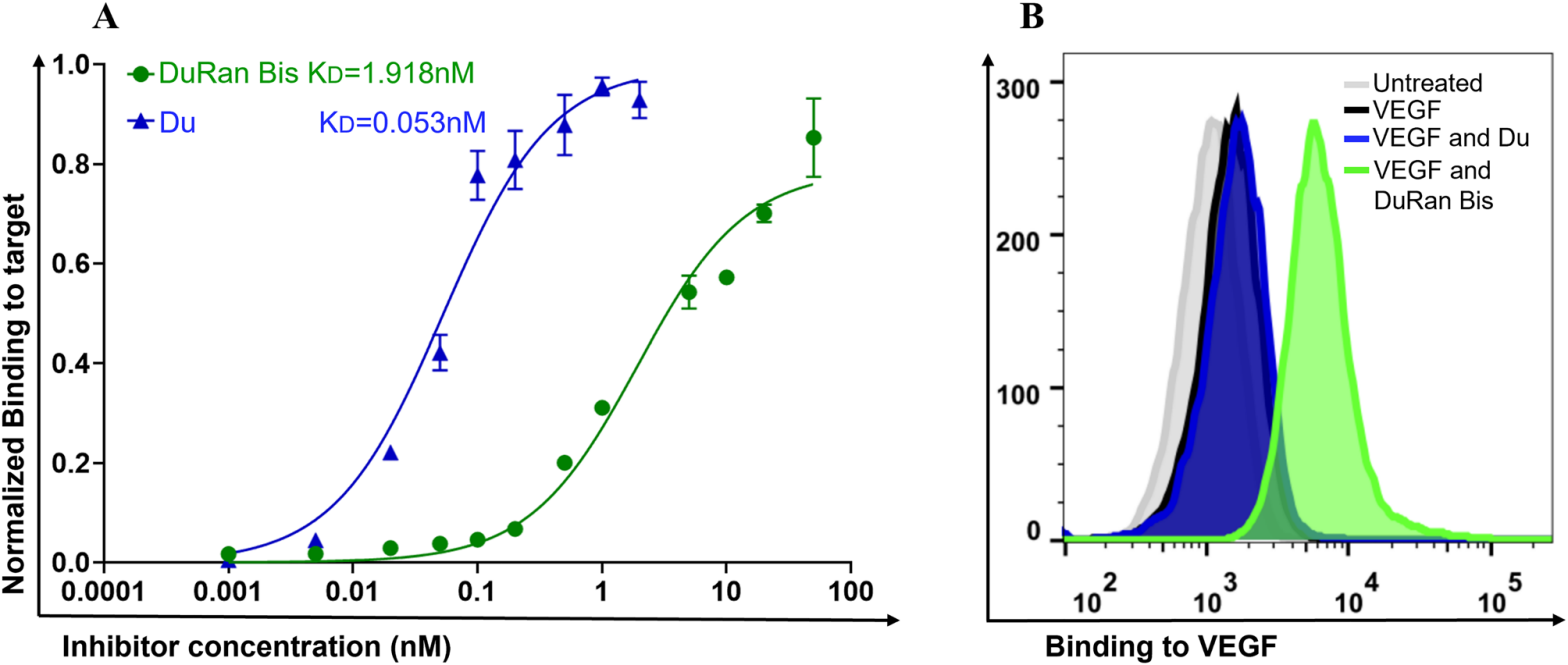
Binding of the mono- and bi-specific proteins to cell-expressed PDL1 and recombinant VEGF_121_. (**A**) Binding titration curves for the Du mono-specific (blue triangles) and DuRan-Bis bi-specific (green circles) proteins to endogenous PDL1 on the surface of U87MG cells. Binding was measured using FACS at protein concentrations of 0.001–5 nM (Du) and 0.001–50 nM (DuRan-Bis). The binding was determined using an APC-conjugated anti-His tag antibody. Fluorescence signals obtained from cells treated with APC-conjugated anti-His tag antibody in the absence of Du or DuRan-Bis proteins were subtracted from all samples (containing different protein treatments), and the resulting signal intensities were then normalized to the highest binding signal exhibited by the same protein treatment. The data was fitted to a 1:1 Langmuir kinetic binding model to yield the apparent K_D_ values for Du and DuRan-Bis proteins. Values are the means of triplicate experiments; bars represent standard error of the mean (SEM). (**B**) Flow cytometry analysis showing the dual binding of the bi-specific protein DuRan-Bis in U87MG cells (that express PDL1) pre-incubated with Du or DuRan-Bis (100 nM) and then monitored to determine the enhancement in binding response upon addition of biotinylated VEGF_121_ (100 nM), followed by streptavidin-conjugated APC. The following color coding is used: grey—untreated cells, black—cells treated with biotinylated VEGF_121_ and then streptavidin-conjugated APC, blue—cells treated with Du followed by biotinylated VEGF_121_ and then streptavidin-conjugated APC, green—cells treated with DuRan-Bis followed by VEGF_121_ and then streptavidin-conjugated APC.

The SPR experiments were complemented by experiments determining the binding affinity of DuRan-Bis to cell-expressed PDL1. For that purpose, U87MG glioblastoma cells, which endogenously express PDL1 on their surface, were used^50,51^. Analysis of the binding titration curve (using a 1:1 Langmuir kinetic binding model) showed that DuRan-Bis binds to U87MG cells with an apparent K_D_ of 1.918 ± 0.290 nM (Fig. 3A). For comparison, determination of the affinity of Du to PDL1 in the same system afforded a K_D_ value of 0.053 ± 0.007 nM. These results are similar to those found in the SPR experiment, where the K_D_ of DuRan-Bis to PDL1 was 0.654 nM and that for Du to PDL1 was 0.057 nM. These slight differences could be due to the more complex environment on the cell surface vs. the chip surface. The higher affinity to PDL1 of Du vs. DuRan-Bis could be due to the larger size of DuRan-Bis and/or to interference in the binding to PDL1 on the cell surface by the other component of DuRan-Bis (the scFv targeting VEGF).

The dual binding capability of the bi-specific binder DuRan-Bis was also demonstrated in cells by pre-incubating the U87MG cells with DuRan-Bis or Du as a control (both at 100 nM) and then monitoring the binding response to Du or DuRan-Bis of biotinylated VEGF_121_ (100 nM), followed by streptavidin-conjugated APC (Fig. 3B). This experiment showed that DuRan-Bis, but not Du, could simultaneously bind both PDL1 and VEGF.

### DuRan-Bis inhibits U87MG cell proliferation

**Figure 4:**
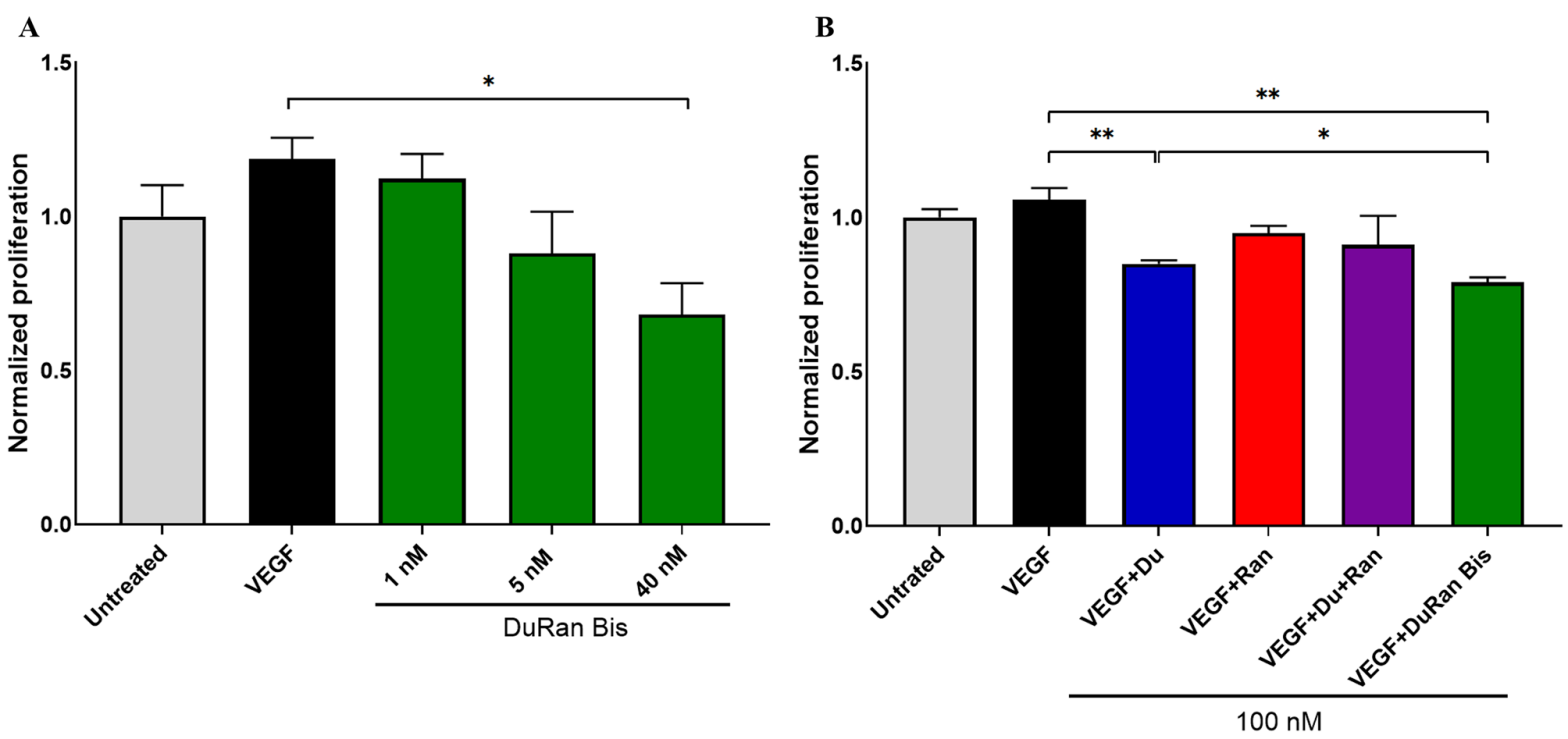
Inhibitory effect of DuRan-Bis on the proliferation of U87MG cells, as shown in an XTT assay. (**A**) U87MG cells were treated for 48 h with 1 nM of VEGF either alone (black) or with 1 nM, 5 nM or 40 nM of DuRan-Bis (green). (**B**) U87MG cells were treated for 48 h with 1 nM of VEGF alone (black) or with 100 nM of Du (blue), Ran (red), a mixture of Du and Ran (purple), or DuRan-Bis (green). Cell viability measured using the XTT reagent was normalized to that of untreated cells (grey). Values are the means of triplicate experiments; bars represent SEM. *P < 0.05; **P < 0.01 (Student’s t-test, compared with cells treated with VEGF alone or with cells treated with VEGF and Du).

To examine the biological activity of DuRan-Bis on cancer cells, we evaluated (using the XTT assay) the ability of DuRan-Bis to inhibit the proliferation of U87MG cells (Fig. 4). To this end, U87MG cells were treated with 1 nM of VEGF_121_ in the absence or presence of 1, 5, or 40 nM DuRan-Bis. Figure 4A shows that increasing concentrations of DuRan-Bis led to a concentration-dependent inhibition of cell proliferation. Samples treated with 40 nM DuRan-Bis/VEGF_121_ showed significantly lower cell proliferation than untreated cells or cells treated solely with VEGF_121_ (1 nM). Previous experiments testing the inhibition of proliferation of U87MG with bevacizumab, a well characterized and FDA approved therapeutic anti-VEGF antibody, showed no inhibition of cell proliferation when low concentrations (in the nanomolar range) of bevacizumab were used, and significant inhibition was seen only for higher bevacizumab concentrations (≥ 3.35 µM)^52,53^. In contrast, DuRan-Bis showed inhibition in the low nano-molar range, which could indicate better inhibition activity of DuRan-Bis compared to bevacizumab. The superior inhibition by DuRan-Bis could be explained by the binding of DuRan-Bis to PDL1, which would increase the local concentration of the inhibitor and hence lead to better inhibition of VEGF_121_.

We then went on to evaluate the inhibitory action of DuRan-Bis on the proliferation of U87MG cells in comparison to Du and Ran mono-specific controls. To this end, U87MG cells were treated with 1 nM of VEGF_121_ alone or VEGF_121_ in the presence of 100 nM Du, Ran, a mixture of Du and Ran, or DuRan-Bis. Treatment with DuRan-Bis resulted in significantly higher inhibition of cell proliferation than treatment with Du or Ran alone or a mixture of the two (Fig. 4B). This superior inhibition potency of the bi-specific protein over its mono-specific counterparts may be the result of binding of DuRan-Bis to PDL1 on the cancer cell surface, thus increasing the local concentration of DuRan-Bis and enabling better inhibition of VEGF. These findings, along with the simultaneous inhibition of both VEGF and PDL1, may explain the superiority of the bi-specific inhibitor over each of the mono-specific inhibitors or their combination. Unexpectedly, Du (targeting cellular PDL1) showed significant inhibition of the proliferation of U87MG cells, albeit slightly less than the bi-specific inhibition. This may explain the fact that for some of the samples treated with VEGF_121_ and the inhibitors, proliferation was lower than that of the untreated cells.

### DuRan-Bis increases immune cell cytotoxicity and reduces SCC47 cell viability

**Figure 5:**
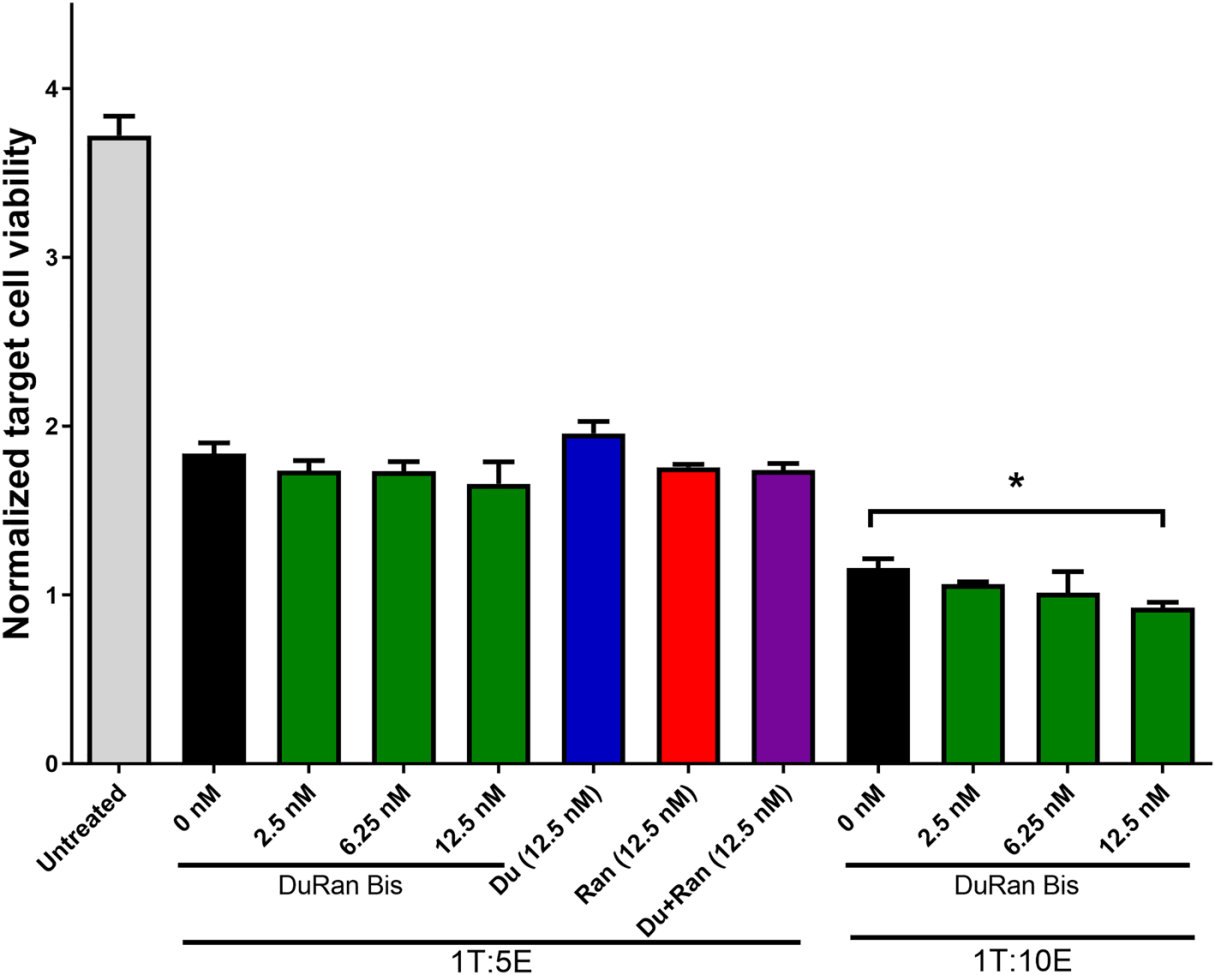
Inhibitory effect of the bi-specific DuRan-Bis on SCC47 cell viability, as determined by using a cytotoxicity assay. SCC47 cells (target cells) were incubated for 48 h together with pre-activated PBMCs (effector cells) in ratios of 1:5 or 1:10 (target to effector cells) either alone (black) or in the presence of 2.5, 6.25, or 12.5 nM of bi-specific DuRan-Bis (green). For the 1:5 ratio, 12.5 nM Du (blue) or Ran (red) alone or in mixture of Du and Ran (purple) were also tested. Analysis of SCC74 cell viability was based on the green fluorescent protein (GFP) content of the cells. Values are the means of triplicate experiments; bars represent SEM. *P < 0.05 (Student’s t-test, compared with cells treated with effector cells alone).

To examine the effect of DuRan-Bis on cancer cell viability and on the function of immune cells, we evaluated the ability of DuRan-Bis to enhance the cytotoxicity of immune cells that destroy SCC47 head and neck cancer cells (Fig. 5). Effector immune cells, i.e., PBMCs extracted from a healthy blood donor, were cultured for 48 h for cell activation. Thereafter, the target cells, i.e., SCC47 cells (that had been seeded into the plates 24 h previously), were treated with 12.5 nM Du, Ran, a mixture of Du and Ran, or DuRan-Bis in the absence of activated effector cells. In addition, different ratios of target to effector cells (i.e., 1:3, 1:5 or 1:10) were left untreated or exposed to 2.5 nM, 6.25 nM, or 12.5 nM DuRan-Bis, and for the target to effector cell ratio of 1:5, treatment with 12.5 nM Du, Ran, or a mixture of Du and Ran was also tested.

Incubation of the target cells with increasing concentrations of DuRan-Bis led to reduced target cell viability in a concentration-dependent manner for ratios of target to effector cells of 1:3 (Supplementary Fig. S4) and 1:5 and 1:10 (Fig. 5) compared to cancer cells treated with effector cells alone (i.e., in the absence DuRan-Bis) or to cancer cells treated with the DuRan-Bis alone (Supplementary Fig. S4), with a significant reduction in cell viability being observed in the 1:10 sample treated with 12.5 nM DuRan-Bis. The lower viability of the target cells treated with effector cells and DuRan-Bis compared to samples treated with effector cells alone or with inhibitors alone (in the absence the effector cells) indicates that DuRan-Bis probably enhances immune cell cytotoxicity.

Samples of SCC47 cells treated with a ratio of 1:5 target to effector cells and 12.5 nM DuRan-Bis showed slightly, but not significantly, lower target cell viability than samples treated with one of the mono-specific proteins (Du or Ran) or with a mixture of the two (Fig. 5). As mentioned above, these differences between the bi-specific inhibitor and the mono-specific inhibitors could be due to an increased local concentration of DuRan-Bis (by virtue of its binding to cell-surface-bound PDL1), which then also inhibits VEGF (known to be secreted from head and neck squamous cell carcinoma cells^54^, including the SCC47 cell line^55^). Since VEGF is known to mediate immunosuppression by several mechanisms, the bi-specific inhibitor may act both directly and indirectly—direct inhibition of T-cell proliferation and functionality (through binding to VEGFR2 expressed on the T cell surface^12,56^) and indirect inhibition of VEGF via enhancement of hypoxia and hence higher effector cell activity^57^. In addition, inhibition of VEGF in the TME will block the enhanced expression of PD1 that leads to CD8 + T cell exhaustion, which impairs their anti-tumor activity^12,56,57^.

### In-vivo toxicity assay

The toxicity of DuRan-Bis was investigated in mice in terms of the change in animal weight (before vs. after treatment for treated and control mice) and differences in liver and kidney metabolites between mice treated with DuRan-Bis and vehicle-treated mice (Supplementary Figs. S5 and S6). Animal weight remained unchanged for seven days for the DuRan-Bis-treated and control mice. Seven days post treatment, the lung and spleen weights of the DuRan-Bis-treated mice were similar to those of the control group, but the kidneys and livers of the DuRan-Bis-treated mice weighed less. However, there were no statistically significant differences between the two groups in liver and kidney metabolites. We may therefore assume that, overall, the toxicity of DuRan-Bis, is very mild, if at all. However, further analyses are needed to establish the toxicity profile of this potential drug.

## Conclusions

With the current understanding that patients with most types of cancer often exhibit a poor response to therapeutics inhibiting a single specific target (i.e., mono-specific therapy), it is believed that drugs that inhibit two different targets simultaneously (i.e., bi-specific therapy) could be a promising alternative to current treatments, i.e., by targeting and inhibiting two different pathways that promote cancer progression, more effective treatment could be achieved. However, bi-specific constructs based on conventional antibodies are difficult to produce, as a result of mispairing of their four polypeptide chains, leading to low yields of the required product. The solution lies in generating bi-specific protein scaffolds from single peptide chains containing binding epitopes for two different targets. Such constructs have the potential to exhibit enhanced inhibition of cancer progression vs. bi-specific antibodies or two mono-specific drugs given together. Bi-specific inhibitors will provide several advantages over two monospecific drugs given together in that they may be less toxic to the patient and cannot give rise to multi-drug interactions, and they will have a single and defined pharmacokinetic profile instead of different pharmacokinetic profiles for each mono-specific drug. In addition, they would be more cost effective than their mono-specific counterparts. Finally, it is predicted that their inhibition activity will be more selective for the target tumor cells than radio- or chemotherapy.

Potential targets for bi-specific therapy are PDL1 and VEGF, which play important roles in immunosuppression and angiogenesis, both being processes that lead to tumor progression. In addition, these two target proteins have been shown to interact with each other, producing a supportive TME. This study showed that PDL1/VEGF dual inhibition could be achieved with a bi-specific inhibitor derived from high-affinity scFvs fused to an Fc fragment, with the bi-specific inhibitor binding simultaneously and with high affinity to both PDL1 and VEGF. Importantly, this bi-specific inhibitor reduced the proliferation U87MG glioblastoma cells with higher inhibitory activity vs. two mono-specific controls given in combination. Furthermore, the bi-specific inhibitor reduced the viability of SCC47 head and neck cancer cells by virtue of enhanced immune cell cytotoxicity conferred by inhibition of PDL1 and VEGF.

Our study thus complements and contributes to ongoing research focused on combined treatments that target the VEGF-VEGFR/PDL1-PD1 axis. These studies include, for example, a bi-specific IgG-scFv treatment in which a full IgG antibody is fused to an scFv domain (e.g., AK112, a PD1/VEGF bispecific protein currently in clinical trials^58^) and a patented PDL1/VEGF bispecific antibody^59^). IgG-derived multi-domain bi-specific proteins such as these could have advantages of stability and long retention times vis-à-vis single domain fragments, such as DuRan-Bis, but during the production and purification process some domain mismatch could occur. An additional disadvantage of IgG-based compounds is that they may be subject to intellectual property barriers (which is less likely for the DuRan-Bis construct). Other bi-specific proteins composed of single chains that have been described in the literature include a diabody (BsDb) comprising two fused single chains targeting VEGF165 and PD1^60^ and a bivalent anti-PD-L1/VEGF nanobody^61^. These proteins are smaller than our bi-specific DuRan-Bis protein, which may be advantageous in terms of tissue penetration. However, the lack of an Fc fragment could constitute a disadvantage in that the Fc fragment serves a double purpose: it retards clearance of the protein from the body (by virtue of increasing protein size and its role in recycling the protein in the blood via the Fc machinery) and it may create dimers that may increase the local concentration of the protein. However, the above studies—while opening important avenues for future research—are still to address a number of issues: For example, even though the bivalent anti-PD-L1/VEGF nanobody inhibited angiogenesis and cell proliferation in vitro, in-vivo analysis is yet to be done. In addition, the BsDb and AK112 treatments target PD1, which is expressed only on T cells, and not PDL1, which is expressed on the target cancer cells (and antigen-presenting cells). We believe that engaging two targets (VEGF and PDL1) that are expressed in the cancer microenvironment may lead to more specific and potent inhibition. Furthermore, we believe that targeting PDL1 will increase the local concentration of the drug on cancer cells, which may lead to higher specific inhibition by VEGF.

The current study has nevertheless some limitations. The first is that it is not based on a number of different cell lines, one of the reasons being that it is difficult to obtain other types of cancer cells with a GFP marker similar to that in the unique head and neck cancer cell line used here (for which cell viability is corelated to GFP expression). Nonetheless, we stress that we did use two different cell lines and in both we showed a reduction in cancer cell viability—results that make our conclusion about the potency of the scFv-based inhibitor stronger and more robust. In addition, two different experimental setups were used to assess the potency of the chimeric molecule, suggesting that our results, being based on more than just a single type of experiment, analysis or cell line, are general. The second limitation is that its effect on normal cells was not evaluated. In the future, we intend to perform additional in vivo studies to test the molecule's toxicity, but this stage is clearly beyond the scope of this study. These in vivo studies will also address the third limitation of a lack of testing of the molecule's potency in preclinical models.

In conclusion, our bi-specific inhibitor represents a novel therapeutic approach for some cancers. Importantly, the dual-specific platform developed can also be applied to generate bi-specific inhibitors for other targets and for other types of cancer.

## Supplementary Information

Supplementary Information.

## Data Availability

The data presented in this study are available on request from the corresponding author.

## References

[1] Sedykh SE, Prinz VV, Buneva VN, Nevinsky GA (2018). Bispecific antibodies: Design, therapy. Perspectives. Drug Des. Devel. Ther..

[2] Runcie K, Budman DR, John V, Seetharamu N (2018). Bi-specific and tri-specific antibodies- the next big thing in solid tumor therapeutics. Mol. Med..

[3] Chames P, Baty D (2009). Bispecific antibodies for cancer therapy: The light at the end of the tunnel?. MAbs.

[4] Schaefer W, Völger HR, Lorenz S, Imhof-jung S, Jörg T, Klein C, Mølhøj M, Schaefer W, Völger HR, Lorenz S (2016). Heavy and light chain pairing of bivalent quadroma and knobs-into-holes antibodies Analyzed by UHR-ESI-QTOF mass spectrometry. MAbs.

[5] Brinkmann U, Kontermann RE (2017). The making of bispecific antibodies. MAbs.

[6] Slade MJ, Uy GL (2020). CD123 Bi-specific antibodies in development in AML: What do we know so far?. Best Pract. Res. Clin. Haematol..

[7] Gunasekaran K, Pentony M, Shen M, Garrett L, Forte C, Woodward A, Ng SB, Born T, Retter M, Manchulenko K (2010). Enhancing antibody Fc heterodimer formation through electrostatic steering effects: Applications to bispecific molecules and monovalent IgG. J. Biol. Chem..

[8] Stieglmaier J, Benjamin J, Nagorsen D (2015). Utilizing the BiTE (bispecific T-cell engager) platform for immunotherapy of cancer. Expert Opin. Biol. Ther..

[9] Zhang X, Yang Y, Fan D, Xiong D (2017). The development of bispecific antibodies and their applications in tumor immune escape. Exp. Hematol. Oncol..

[10] Czajkowsky DM, Hu J, Shao Z, Pleass RJ (2012). Fc-fusion proteins: New developments and future perspectives. EMBO Mol. Med..

[11] Weizman T, Levin I, Zaretsky M, Sagi I, Aharoni A (2017). Increased potency of a bi-specific TL1A-ADAM17 (TACE) inhibitor by cell surface targeting. Front. Mol. Biosci..

[12] Ziogas AC, Gavalas NG, Tsiatas M, Tsitsilonis O, Politi E, Terpos E, Rodolakis A, Vlahos G, Thomakos N, Haidopoulos D (2012). VEGF directly suppresses activation of T cells from ovarian cancer patients and healthy individuals via VEGF receptor type 2. Int. J. Cancer.

[13] Meder L, Schuldt P, Thelen M, Schmitt A, Dietlein F, Klein S, Borchmann S, Wennhold K, Vlasic I, Oberbeck S (2018). Combined VEGF and PD-L1 blockade displays synergistic treatment effects in an autochthonous mouse model of small cell lung cancer. Cancer Res..

[14] Yi M, Jiao D, Qin S, Chu Q, Wu K, Li A (2019). Synergistic effect of immune checkpoint blockade and anti-angiogenesis in cancer treatment. Mol. Cancer.

[15] Xue S, Hu M, Li P, Ma J, Xie L, Teng F (2017). Relationship between expression of PD-L1 and angiogenesis, proliferation, and invasion in glioma tumor. Oncotarget.

[16] Shin S, Jeon YK, Kim P, Cho YM (2016). Clinicopathologic analysis of PD-L1 and PD-L2 expression in renal cell carcinoma: Association with oncogenic proteins status. Ann. Surg. Oncol..

[17] Gerber, H., Baldwin, M.E., Shojaei, F. Vascular endothelial growth factor antibodies for anti-angiogenic therapy. In *Tumor Angiogenesis*; Springer, Berlin, Heidelberg, 2008; pp. 377–393.

[18] Kaiser PK, Chung CY, Ph D, Kim RY (2006). Study, M. Ranibizumab for neovascular age-related macular degeneration. N. Engl. J. Med..

[19] Pàez-ribes M, Allen E, Hudock J, Takeda T, Viñals F, Inoue M, Bergers G, Hanahan D (2009). Antiangiogenic therapy elicits malignant progression of tumors to increased local invasion and distant metastasis. Cancer Cell.

[20] Narayana A, Kunnakkat SD, Medabalmi P, Golfinos J, Parker E, Knopp E, Zagzag D, Eagan P, Gruber D, Gruber ML (2012). Change in pattern of relapse after antiangiogenic therapy in high-grade glioma. Int. J. Radiat. Oncol. Biol. Phys..

[21] Huang Y, Kim BYS, Chan CK, Hahn SM, Weissman IL, Jiang W (2018). Improving immune-vascular crosstalk for cancer immunotherapy. Nat. Rev. Immunol..

[22] Mcdermott DF, Sosman JA, Sznol M, Massard C, Gordon MS, Hamid O, Powderly JD, Infante JR, Fasso M, Wang YV (2016). Atezolizumab, an anti – programmed death-ligand 1 antibody, in metastatic renal cell carcinoma: Long-term safety, clinical activity, and immune correlates from a phase Ia study. J. Clin. Oncol..

[23] Apolo AB, Infante JR, Balmanoukian A, Patel MR, Wang D, Kelly K, Mega AE, Britten CD, Ravaud A, Mita AC (2017). Avelumab, an anti – programmed death-ligand 1 antibody, in patients with refractory metastatic urothelial carcinoma : Results from a multicenter. Phase Ib Study. J. Clin. Oncol..

[24] Hui R, Ph D, Yokoi T, Ph D, Chiappori A, Lee KH, Ph D, Wit M, Cho BC (2017). Durvalumab after chemoradiotherapy in stage III non– small-cell lung cancer. N. Engl. J. Med..

[25] Zou W, Wolchok JD, Chen L (2016). PD-L1 ( B7–H1) and PD-1 pathway blockade for cancer therapy: Mechanisms, response biomarkers, and combinations. Sci. Transl. Med..

[26] Faiena I, Cummings AL, Crosetti AM, Pantuck AJ, Chamie K, Drakaki A (2018). Durvalumab: An investigational anti-PD-L1 monoclonal antibody for the treatment of urothelial carcinoma. Drug Des. Devel. Ther..

[27] Shen X, Zhao B (2018). Efficacy of PD-1 or PD-L1 inhibitors and PD-L1 expression status in cancer: Meta-analysis. BMJ.

[28] Alsaab HO, Sau S, Alzhrani R, Tatiparti K, Bhise K, Kashaw SK, Iyer AK (2017). PD-1 and PD-L1 checkpoint signaling inhibition for cancer immunotherapy: Mechanism, combinations, and clinical outcome. Front. Pharmacol..

[29] Georganaki M, van Hooren L, Dimberg A (2018). Vascular targeting to increase the efficiency of immune checkpoint blockade in cancer. Front. Immunol..

[30] Huang Y, Yuan J, Righi E, Kamoun WS, Ancukiewicz M, Nezivar J, Santosuosso M, Martin JD, Martin MR, Vianello F (2012). Vascular normalizing doses of antiangiogenic treatment reprogram the immunosuppressive tumor microenvironment and enhance immunotherapy. Proc. Natl. Acad. Sci. U. S. A..

[31] Väyrynen JP, Tuomisto A, Klintrup K, Mäkelä J, Karttunen TJ, Mäkinen MJ (2013). Detailed analysis of inflammatory cell infiltration in colorectal cancer. Br. J. Cancer.

[32] O’Donnell JS, Long GV, Scolyer RA, Teng MWL, Smyth MJ (2017). Resistance to PD1 / PDL1 checkpoint inhibition. Cancer Treat. Rev..

[33] Puccini A, Battaglin F, Iaia ML (2020). Overcoming resistance to anti- ­ PD1 and L1 treatment in gastrointestinal malignancies. J. Immunother. Cancer.

[34] Socinski MA, Jotte RM, Cappuzzo F, Orlandi F, Stroyakovskiy D, Nogami N, Rodríguez-Abreu D, Moro-Sibilot D, Thomas CA, Barlesi F (2018). Atezolizumab for first-line treatment of metastatic nonsquamous NSCLC. N. Engl. J. Med..

[35] Quintela-Fandino M, Holgado E, Manso L, Morales S, Bermejo B, Colomer R, Apala JV, Blanco R, Muñoz M, Caleiras E (2020). Immuno-priming durvalumab with bevacizumab in HER2-negative advanced breast cancer: A pilot clinical trial. Breast Cancer Res..

[36] Cui X, Jia H, Xin H, Zhang L, Chen S, Xia S, Li X, Xu W, Chen X, Feng Y (2021). A novel bispecific antibody targeting PD-L1 and VEGF with combined anti-tumor activities. Front. Immunol..

[37] Chao G, Lau WL, Hackel BJ, Sazinsky SL, Lippow SM, Wittrup KD (2006). Isolating and engineering human antibodies using yeast surface display. Nat. Protoc..

[38] Badarni M, Prasad M, Golden A, Bhattacharya B, Levin L, Yegodayev KM, Dimitstein O, Joshua BZ, Cohen L, Khrameeva E (2021). Igf2 mediates resistance to isoform-selective-inhibitors of the Pi3k in Hpv positive head and neck cancer. Cancers.

[39] Hunter SA, Cochran JR (2016). Cell-binding assays for determining the affinity of protein-protein interactions: Technologies and considerations.

[40] Badarni M, Prasad M, Balaban N, Zorea J, Yegodayev KM, Joshua BZ, Dinur AB, Grénman R, Rotblat B, Cohen L (2019). Repression of AXL expression by AP-1/JNK blockage overcomes resistance to PI3Ka therapy. JCI Insight.

[41] Kythreotou A, Siddique A, Mauri FA, Bower M, Pinato DJ (2018). PD-L1. Journael Clin. Pathol..

[42] Blick SKA, Keating GM, Wagstaff AJ (2007). Ranibizumab. Drugs.

[43] Cembrola B, Ruzza V, Troise F, Esposito ML, Sasso E, Cafaro V, Passariello M, Visconte F, Raia M, Del Vecchio L (2019). Rapid affinity maturation of novel anti-PD-L1 antibodies by a fast drop of the antigen concentration and FACS selection of yeast libraries. Biomed Res. Int..

[44] Lee HT, Lee JY, Lim H, Lee SH, Moon YJ, Pyo HJ, Ryu SE, Shin W, Heo Y-S (2017). Molecular mechanism of PD-1 / PD-L1 blockade via anti-PD-L1 antibodies Atezolizumab and Durvalumab. Sci. Rep..

[45] Oren O, Banerjee V, Taube R, Papo N (2018). An Aβ42 variant that inhibits intra- and extracellular amyloid aggregation and enhances cell viability. Biochem. J..

[46] Lowe J, Araujo J, Yang J, Reich M, Oldendorp A, Shiu V, Quarmby V, Lowman H, Lien S, Gaudreault J (2007). Ranibizumab inhibits multiple forms of biologically active vascular endothelial growth factor in vitro and in vivo. Exp. Eye Res..

[47] Schofield DJ, Percival-alwyn J, Rytelewski M, Hood J, Rothstein R, Wetzel L, Mcglinchey K, Adjei G, Machiesky L, Chen W (2021). Activity of murine surrogate antibodies for Durvalumab and Tremelimumab lacking effector function and the ability to deplete regulatory T cells in mouse models of cancer. MAbs.

[48] Teran M, Nugent MA (2019). Characterization of receptor binding kinetics for vascular endothelial growth factor-A using SPR. Anal. Biochem..

[49] Lee HT, Lee SH, Heo YS (2019). Molecular interactions of antibody drugs targeting PD-1, PD-L1, and CTLA-4 in immuno-oncology. Molecules.

[50] Peng H, Li Z, Fu J, Zhou R (2019). Growth and differentiation factor 15 regulates PD-L1 expression in glioblastoma. Cancer Manag. Res..

[51] Su L, Guo W, Lou L, Nie S, Zhang Q, Liu Y, Chang Y, Zhang X, Li Y, Shen H (2020). EGFR-ERK pathway regulates CSN6 to contribute to PD-L1 expression in glioblastoma. Mol. Carcinog..

[52] Mesti T, Savarin P, Triba MN, Moyec Le L, Ocvirk J, Banissi C, Carpentier AF (2014). Metabolic impact of anti-angiogenic agents on U87 glioma cells. PLoS One.

[53] Huang HE, Song J, Liu Z, Pan LI, Xu G (2018). Autophagy activation promotes bevacizumab resistance in glioblastoma by suppressing Akt/MTOR signaling pathway. Oncol. Lett..

[54] O-charoenrat P, Rhys-Evans P, Eccles SA (2001). Expression of vascular endothelial growth factor family members in head and neck squamous cell carcinoma correlates with lymph node metastasis. Cancer.

[55] Ludwig N, Yerneni SS, Razzo BM, Whiteside TL (2018). Exosomes from HNSCC promote angiogenesis through reprogramming of endothelial cells. Mol. Cancer Res..

[56] Gavalas NG, Tsiatas M, Tsitsilonis O, Politi E, Ioannou K, Ziogas AC, Rodolakis A, Vlahos G, Thomakos N, Haidopoulos D (2012). VEGF directly suppresses activation of T cells from ascites secondary to ovarian cancer via VEGF receptor type 2. Br. J. Cancer.

[57] De Almeida PE, Mak J, Hernandez G, Jesudason R, Herault A, Javinal V, Borneo J, Kim JM, Walsh KB (2020). Anti-VEGF treatment enhances CD8+T-cell antitumor activity by amplifying hypoxia. Cancer Immunol. Res..

[58] Wu L, Li G, Xia B (2021). 430 A phase 1b/II clinical study of AK112, a PD-1/VEGF bispecific antibody, in combination with Olaparib in BRCA germline wild-type platinum sensitive recurrent ovarian cancer. J. Immunother. Cancer.

[59] Wang, Z., Chen, Y., Li, D., Li, J. A bispecific anti-PD-L1/VEGF antibody and uses there of 2021.

[60] Xiong C, Mao Y, Wu T, Kang N, Zhao M, Di R, Li X, Ji X, Liu Y (2018). Optimized expression and characterization of a novel fully human bispecific single-chain diabody targeting vascular endothelial growth factor165 and programmed death-1 in Pichia pastoris and evaluation of antitumor activity in vivo. Int. J. Mol. Sci..

[61] Hassanzadeh Eskafi A, Oghalaei A, Mahboudi F, Ghaderi H, Behdani M, Shoari A, Kazemi-Lomedasht F (2023). Investigation of the therapeutic potential of recombinant bispecific bivalent anti-PD-L1/VEGF nanobody in inhibition of angiogenesis. Immunopharmacol. Immunotoxicol..

