# Supplementary material for: Developing a dual VEGF/PDL1 inhibitor based on high-affinity scFv heterodimers as an anti-cancer therapeutic strategy
Source: Sci Rep. 2023 Jul 24;13:11923. doi: 10.1038/s41598-023-39076-8 (PMC10366146; doi:10.1038/s41598-023-39076-8)
Supplement: Supplementary file 1 — Supplementary Information. [file 41598_2023_39076_MOESM1_ESM.docx]

**Supplementary Material**

Developing a Dual VEGF/PDL1 Inhibitor Based on High‑Affinity scFv Heterodimers as an Anti-cancer Therapeutic Strategy

Noam Tzuri, Ksenia M. Yegodayev, Ofra Novoplansky, Moshe Elkabets, Amir Aharoni, Niv Papo


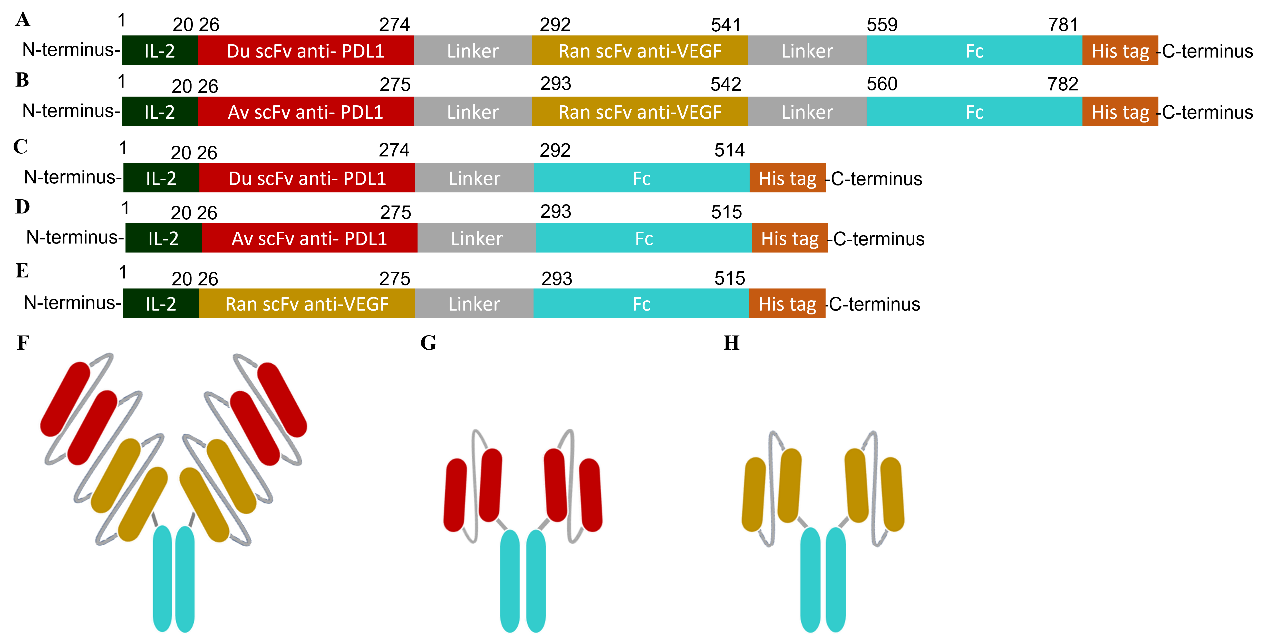


**Figure S1. Bi- and mono-specific protein constructs. (A-E)** Linear illustrations of the bi-specific proteins DuRan-Bis (A) and AvRan-Bis (B), the anti-PDL1 mono-specific protein Du or Av (C,D), and the anti-VEGF mono-specific protein Ran (E). All the constructs include an N-terminal IL-2 signal peptide (MYRMQLLSCIALSLALVTNS) (residues 1-20). (A,B) The bi-specific construct DuRan-Bis or AvRan-Bis includes: the Du or Av scFv, which is derived from an anti-PDL1 antibody (residues 26-274/275 for DuRan-Bis or AvRan-Bis, respectively); a flexible linker (GGGGSGGGGSGGGGS); the Ran scFv, which is derived from an anti-VEGF antibody (residues 292/293-541/542 for DuRan-Bis or AvRan-Bis, respectively); a flexible linker (GGGGSGGGGSGGGGS); a human IgG1 Fc domain (residues 559/560-781/782 for DuRan-Bis or AvRan-Bis, respectively); and a C-terminal 6×His tag. (C) The Du construct includes the Du scFv, which is derived from an anti-PDL1 antibody (residues 26-274); a flexible linker (GGGGSGGGGSGGGGS); a human IgG1 Fc domain (residues 292-514); and a C-terminal 6×His tag. (D) The Av construct includes the Av scFv, which is derived from an anti-PDL1 antibody (residues 26-275); a flexible linker (GGGGSGGGGSGGGGS); a human IgG1 Fc domain (residues 293-515); and a C-terminal 6×His tag. (E) The Ran construct includes the Ran scFv, which is derived from an anti-VEGF antibody (residues 26-275); a flexible linker (GGGGSGGGGSGGGGS); a human IgG1 Fc domain (residues 293-515); and a C-terminal 6×His tag. **(F, G, H)** Structural illustrations of the bi-specific protein (F), the anti-PDL1 mono-specific protein (G), and the anti-VEGF mono-specific protein (G).


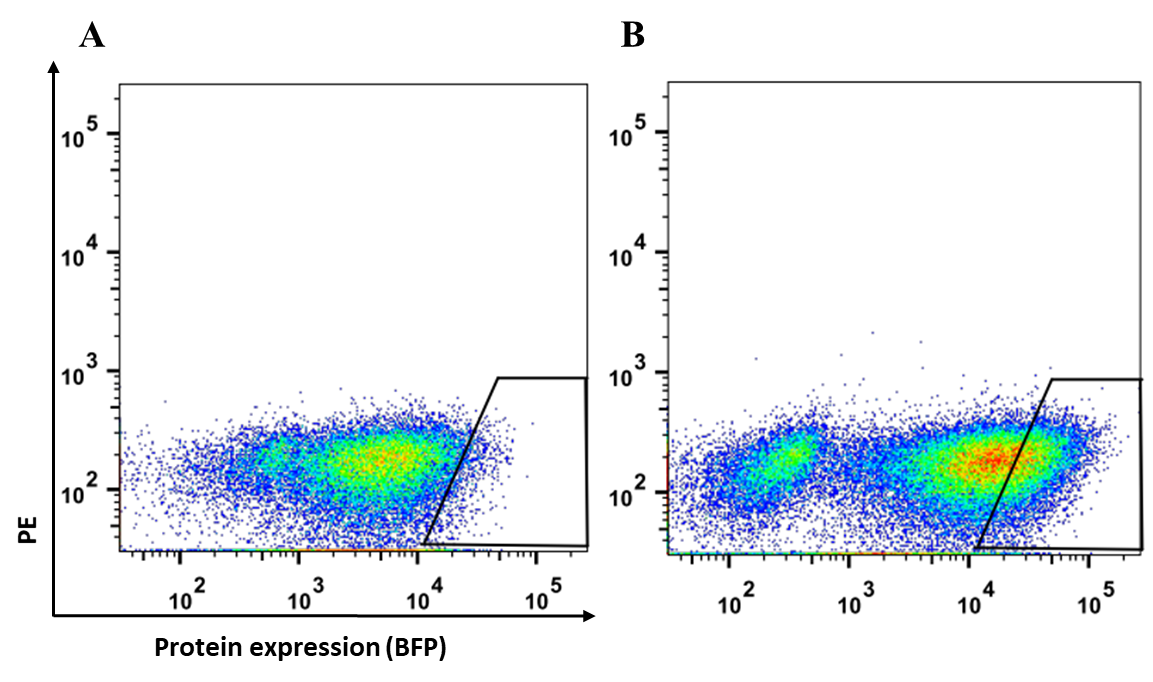


**Figure S2. FACS sorting of HEK293T cells overexpressing the AvRan-Bis and DuRan-Bis proteins**. (**A,B**) HEK293T cells with high expression levels of BFP protein were sorted using a black polygon-shaped gate. (A) Cells expressing the AvRan-Bis protein. (B) Cells expressing the DuRan-Bis protein.

**DuRan Bis**

**Du**

**Ran**

**M**

**100 kDa**

**75 kDa**

**180 kDa**

**140 kDa**

**60 kDa**

**45 kDa**

**35 kDa**

**Figure S3. SDS-PAGE analysis of the purified mono- and bi-specific proteins.** Bi-specific DuRan-Bis protein and mono-specific Du and Ran proteins under reducing conditions. M -protein marker.


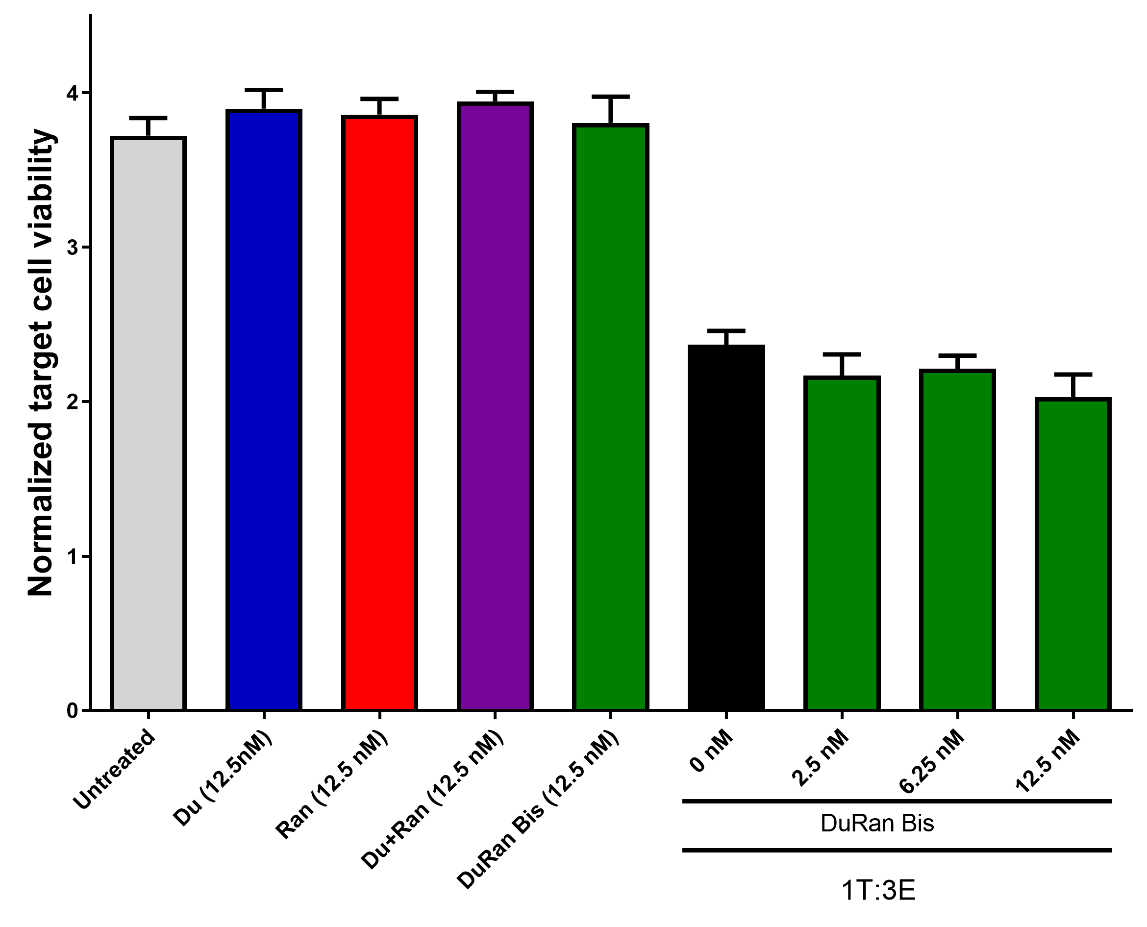


**Figure S4. Inhibitory effect of the bi-specific DuRan Bis chimera on the viability of SCC47 cells, as determined by a cytotoxicity assay.** SCC47 cells (target cells) were treated with pre-activated PBMC (effector cells) at a ratio of 1:3 target to effector cells either in the absence (black) or in the presence of 2.5, 6.25, or 12.5 nM bi-specific DuRan-Bis (green). Target calls were also treated with 12.5 nM DuRan-Bis (green), Du (blue) or Ran (red), each alone, or with a mixture of Du and Ran (purple) in the absence of PBMC cells (gray). The different treatments were incubated with the target cells for 48 h. Analysis of SCC47 cell viability was based on the green fluorescent protein (GFP) content of the cells. Bars represent the standard error of the mean (SEM) of triplicate experiments. *P < 0.05 (Student’s t-test, compared with cells exposed to effector cells alone, untreated cells, or the Ran mono-specific treatment)**.**

**
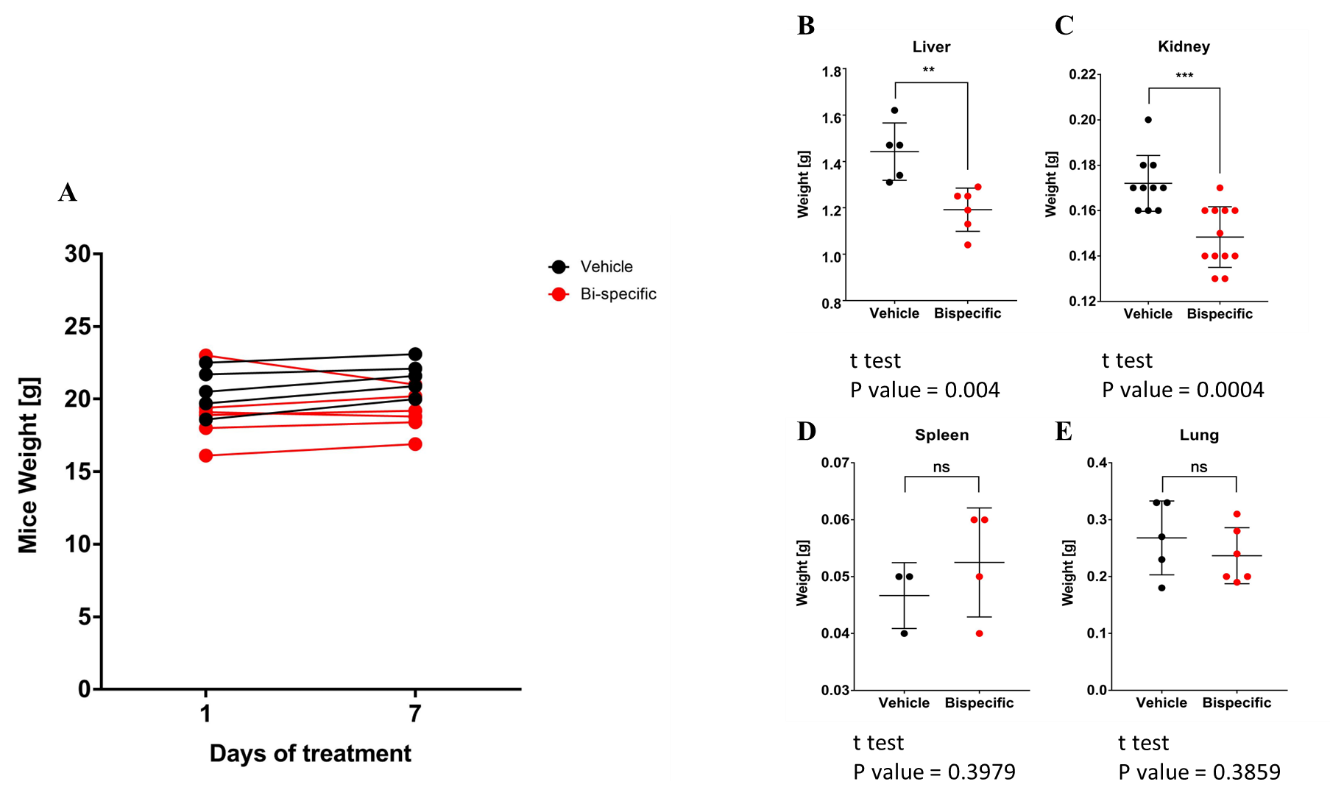
**

**Figure S5. Effect of the bispecific** **DuRan-Bis protein on total weight and individual organ weight in mice. (A)** Total weight on the day of treatment with DuRan-Bis (1.25 mg/kg, n = 6) or PBS (vehicle, n=5) and 7 days post treatment. **(B-E)** Weights of individual organs 7 days post treatment: (B) Liver, (C) Kidneys, (D) Spleen and (E) Lungs. *P < 0.05 (Student’s t-test)**.**


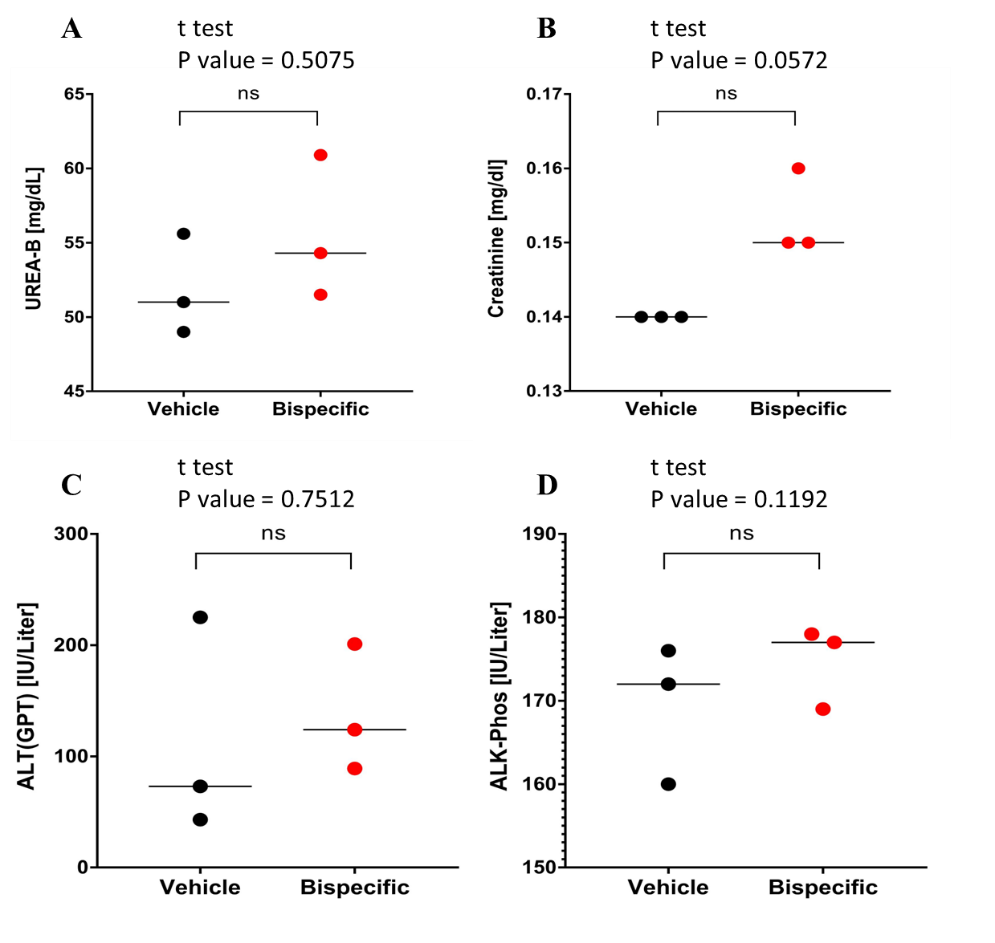


**Figure S6. Effect of DuRan-Bis on kidney and liver activity in mice. (A-D)** Kidney and liver metabolites were determined 7 days post treatment with DuRan-Bis (1.25 mg/kg, n=6) or PBS control (vehicle, n=5). For the treated group, serum from two mice was pooled (n=3) for biochemistry analysis. **(A, B)** Serum levels of kidney metabolites: (A) urea-B and (B) creatinine. **(C, D)** Serum levels of liver metabolites: (C) alanine transaminase (ALT) and (D) alkaline phosphatase (ALK-Phos). *P < 0.05 (Student’s t-test)**.**

**Table S1**. Primers used in the cloning stage for the generation of the Du, Ran, Av, AvRan-Bis and DuRan-Bis genes. Non-bold letters indicate homology to the amplified gene and bold letters indicate homology to plasmid/addition. F, forward primer; R, reverse primer.

| Primer reverse 5' -> 3' | Primer forward 5' -> 3' |  |  |
| --- | --- | --- | --- |
| **CCATGGCCGATATCGAATTCGTGAC** | **AGATCTGTGGAGTGCCCACCTTG** | Opening the plasmid for Gibson | A |
| **GGATCCACCGCCTCCAGATCCGCCTCCACCGCTGCCCCCTCCGCC**  GCTTGACACGGTCACTAGGGTTCC | **CTTGCACTTGTCACGAATTCGATATCGGCCATGGAT**CAGTCTGCCCTTACGCAGCCAGCC | Avelumab scFv first amplification with pFUSE homology (F) and linker adding (R) | B1 |
| **GGATCCACCGCCTCCAGATCCGCCTCCACCGCTGCCCCCTCCGCC**GCTACTAACCGTGACGAGTGTGCC | **CTTGCACTTGTCACGAATTCGATATCGGCCATGGAT**GAAATCGTGCTTACACAGAGCCCTGGAAC | Durvalumab scFv first amplification with pFUSE homology (F) and linker adding (R) | B2 |
| **GGATCCACCGCCTCCAGATCCGCCTCCACCGCTGCCCCCTCCGCC**  ACTAGACACGGTCACCAGGGTGC | **CTTGCACTTGTCACGAATTCGATATCGGCCATGGAT**GATATTCAGCTGACCCAGTCTCCG | Ranibizumab scFv first amplification with pFUSE homology (F) and linker adding (R) | B3 |
| **GTGCTGGGCAAGGTGGGCACTCCACAGATCT**GGATCCACCGCCTCCAGATCCG | **CTTGCACTTGTCACGAATTCGATATCGGCCATGGAT**CAGTCTGCCCTTACGCAGCCAGCC | Avelumab scFv second amplification with pFUSE homology (F) and Fc homology (R) | C1 |
| **GTGCTGGGCAAGGTGGGCACTCCACAGATCT**GGATCCACCGCCTCCAGATCCG | **CTTGCACTTGTCACGAATTCGATATCGGCCATGGAT**GAAATCGTGCTTACACAGAGCCCTGGAAC | Durvalumab scFv second amplification with pFUSE homology (F) Fc homology (R) | C2 |
| **GTGCTGGGCAAGGTGGGCACTCCACAGATCT**GGATCCACCGCCTCCAGATCCG | **CTTGCACTTGTCACGAATTCGATATCGGCCATGGAT**GATATTCAGCTGACCCAGTCTCCG | Ranibizumab scFv second amplification with pFUSE homology (F) and Fc homology (R) | C3 |
| **AGGATTCAAGTAGAGGCTTGATTTGGAGG** | **GTTTTCTGTTCTGCGCCGTTACAG** | Colony-PCR validation | D |
| **GGCCGATATCGAATTCGTGACAAGTG** | **ATGGATGATATTCAGCTGACCCAGTCTC** | Amplification of the plasmid contain ranibizumab scFv for opening | E |
| **GGAGACTGGGTCAGCTGAATATC**ATCCATGGATCCACCGCCTCCAGATCCGCCTCCACCGCTGC | **CTTGCACTTGTCACGAATTCGATATCGGCCATGGAT**CAGTCTGCCCTTACGCAGCCAGCC | Avelumab scFv amp with pFUSE homology (F) and ranibizumab homology (R) | F1 |
| **GGAGACTGGGTCAGCTGAATATC**ATCCATGGATCCACCGCCTCCAGATCCGCCTCCACCGCTGC | **CTTGCACTTGTCACGAATTCGATATCGGCCATGGAT**GAAATCGTGCTTACACAGAGCCCTGGAAC | Durvalumab scFv amp with pFUSE homology (F) ranibizumab homology (R) | F2 |
| **TAGGGGGGGGGGAGGGATCC**TCAGTGATGGTGATGGTGATGTTTACCC | **ACTAGAGAACCAGCGGCCGC**ATGTACAGGATGCAACTCCTGTCTTGC | IL-2 and Bi-specific construct amp with pHAGE homology (F) and IRES homology (R) | G1 |
